# Diet, exercise, and supplements: what is their role in the management of the metabolic dysfunction-associated steatotic liver disease in children?

**DOI:** 10.1007/s12020-024-03783-7

**Published:** 2024-03-22

**Authors:** Anastasios Serbis, Stergios A. Polyzos, Stavroula A. Paschou, Ekaterini Siomou, Dimitrios N. Kiortsis

**Affiliations:** 1 Department of Pediatrics, School of Medicine, University of Ioannina, Ioannina, Greece; 2 First Laboratory of Pharmacology, School of Medicine, Aristotle University of Thessaloniki, Thessaloniki, Greece; 3 Endocrine Unit and Diabetes Center, Department of Clinical Therapeutics, Alexandra Hospital, School of Medicine, National and Kapodistrian University of Athens, Athens, Greece; 4 Laboratory of Physiology, Medical School, University of Ioannina, Ioannina, Greece

**Keywords:** NAFLD, Children, Diet, Exercise, Lifestyle interventions, Dietary supplements

## Abstract

Metabolic dysfunction-associated steatotic liver disease (MASLD), previously known as nonalcoholic fatty liver disease (NAFLD), is the main cause of chronic liver disease in children and adolescents. Indeed, epidemiological studies have shown that MASLD affects up to 40% of children with obesity. Despite the recent approval of medications that target weight loss in adolescents that could have benefits on pediatric MASLD, lifestyle interventions, such as diet and exercise, remain the mainstay of our therapeutic approach. More specifically, studies on diet alone have focused on the possible role of carbohydrate or fat restriction, albeit without a definite answer on the best approach. Weight loss after dietary intervention in children with obesity and MASLD has a beneficial effect, regardless of the diet used. In relation to the role of exercise in MASLD reversal, indirect evidence comes from studies showing that a sedentary lifestyle leading to poor fitness, and low muscle mass is associated with MASLD. However, research on the direct effect of exercise on MASLD in children is scarce. A combination of diet and exercise seems to be beneficial with several studies showing improvement in surrogate markers of MASLD, such as serum alanine aminotransferase and hepatic fat fraction, the latter evaluated with imaging studies. Several dietary supplements, such as vitamin E, probiotics, and omega-3 fatty acid supplements have also been studied in children and adolescents with MASLD, but with equivocal results. This review aims to critically present available data on the effects of lifestyle interventions, including diet, exercise, and dietary supplements, on pediatric MASLD, thus suggesting a frame for future research that could enhance our knowledge on pediatric MASLD management and optimize clinicians’ approach to this vexing medical condition.

## Introduction

Metabolic dysfunction-associated steatotic liver disease (MASLD), formerly known as nonalcoholic fatty liver disease (NAFLD) [1], is the most common cause of chronic liver disease in pediatric populations, more frequently diagnosed in male children with obesity [2]. MASLD encompasses conditions with varying severity, ranging from simple steatosis, which is increased hepatic fat accumulation without inflammation, to the so-called metabolic dysfunction-associated steatohepatitis (MASH), known as nonalcoholic steatohepatitis (NASH). MASH is characterized by increased liver fat accompanied by inflammation and hepatocellular injury, with or without liver fibrosis. MASH progression is not well characterized in children, but it may lead to extensive bridging fibrosis, cirrhosis, and finally, liver failure in early adulthood in a minority of cases [3].

MASLD usually coexists with metabolic syndrome components, such as central adiposity, dyslipidemia, hyperglycemia, and insulin resistance. In addition, several studies have shown that increased liver fat content is associated with an adverse cardiometabolic risk profile already in childhood, adolescence, and early adulthood [4–6]. During the last two decades, the exponential increase in the prevalence of pediatric obesity led to an increase in MASLD prevalence as well. Recent studies have shown that MASLD prevalence is between 5% and 10% in the general pediatric population [2], and almost 40% among children with obesity [7]. Even more alarming is the pace at which both the incidence and the prevalence of the disease have been increasing during the last decades [8, 9], rendering the management of children with MASLD a challenging issue. However, no approved pharmacological therapies exist. The most recent guidelines by both the European Society for Pediatric Gastroenterology, Hepatology and Nutrition (ESPGHAN) [10], and the North American Society of Pediatric Gastroenterology, Hepatology and Nutrition (NASPGHAN) [11], advocate the use of lifestyle interventions, including diet and exercise, as therapeutic measures for pediatric MASLD. Nevertheless, no detailed recommendations exist regarding either the ideal diet, or the type, intensity, and duration of exercise that should be followed by the patients, probably because relevant data are scarce. In addition, studies in adults with MASLD have shown that, among dietary supplements, vitamin E may lead to the improvement of liver function tests (LFTs), hepatic steatosis and inflammation [12–15]. Data on dietary supplements in children with MASLD are even more controversial [16–19].

The current review focused on clinical studies that have examined the efficacy of lifestyle interventions, namely diet and exercise, alone or in combination, in the management of MASLD in children and adolescents. Studies on dietary supplements in children with the disease were also included. It should be noted that although the terms MASLD and NAFLD are used interchangeably, the criteria for the two diagnoses are not entirely the same [1]. Some very recent studies showed a high concordance between the two diagnoses in adults (98%) [20], but lower in children (≥75%) [21]. Until more data are available regarding the concordance of the two terms, we maintained NAFLD nomenclature for earlier studies that used the prior NAFLD definition, throughout our manuscript.

## Materials and methods

Although this is a narrative review, literature was searched according to the Preferred Reporting Items for Systematic Reviews and Meta-Analyses (PRISMA) guidelines [22]. A literature search was conducted on PubMed/Medline database for studies from peer-reviewed journals, published between January 1, 1980, and July 31, 2023, using the following key words: “NAFLD”, “non-alcoholic fatty liver disease”, “MASLD”, “metabolic dysfunction-associated steatotic liver disease”, “fatty liver”, “hepatic steatosis”, “nutrition”, “diet”, “exercise”, “physical activity”, “lifestyle interventions”, “dietary supplements”. Studies in English and in pediatric populations were included. Case reports, reviews, editorials, letters to the editor, case-control and cross-sectional studies were excluded from the review. Relevance was initially screened according to title and abstract. At the stage of eligibility, full-text articles of all relevant studies were reviewed. Papers that were identified from the reference lists of the retrieved articles, were also considered.

**Fig. 1:**
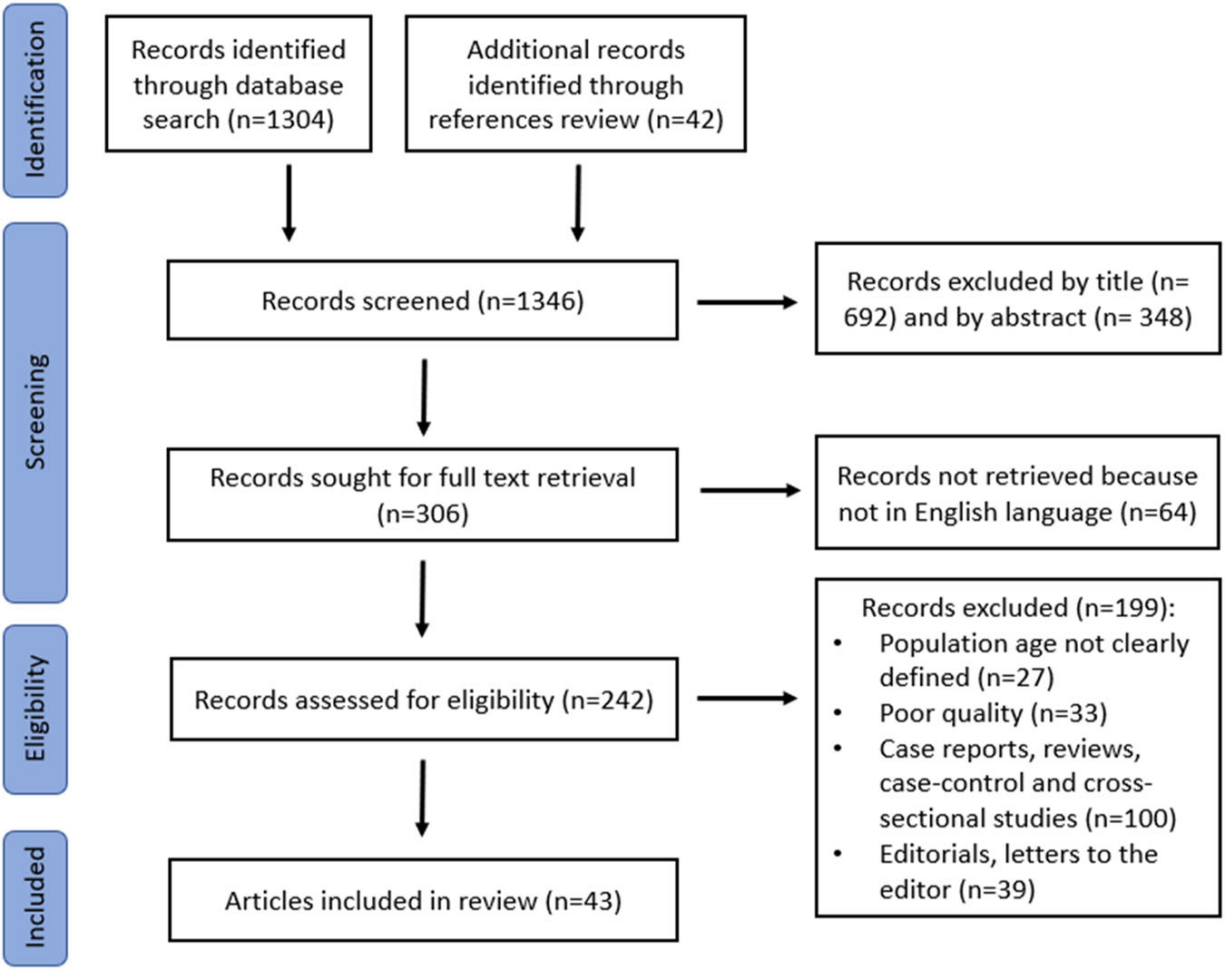
PRISMA flowchart indicating the process for identification and selection of the included studies

Initial literature search yielded 1304 records of which, 692 were excluded by title and 348 by abstract. In addition, 64 records were excluded because they were not written in English. From the reference list of the retrieved reports, 42 additional records were considered relevant. Among the 242 reports that were retrieved and reviewed in full-text, 199 were excluded for various reasons (Fig. 1). In the end, 43 articles were considered pertinent and were included in the current review.

## Results

### Dietary interventions

Five studies were found that evaluated the efficacy of diet intervention on NAFLD parameters in children and adolescents (Table 1). The earliest of these studies in 2009 by Vos et al. [23] was a randomized controlled trial (RCT) comprising 10 children with NAFLD. Seven of these children had a liver biopsy demonstrating NASH, while the other three were diagnosed by LFTs and ultrasonography. Six children were assigned to a low-fructose diet, while the other four to a low-fat diet, according to the American Heart Association recommendations [24]. A significant change in alanine aminotransferase (ALT) was not demonstrated in either group. No follow-up biopsy was performed in any of the participants. Low fructose diet was evaluated in another study by Jin et al. comprising 24 overweight Hispanic American adolescents with increased hepatic fat measured by magnetic resonance spectroscopy (MRS) at baseline [25]. These adolescents were randomized to calorie matched fructose-only or glucose-only beverages provided by the study. No other diet or physical activity modification was implemented. After four weeks, no measurable improvement in hepatic steatosis was identified, estimated by a follow-up MRS. However, several factors related to cardiovascular disease were improved, such as insulin sensitivity of adipose tissue, high sensitivity C-reactive protein (hs-CRP) and oxidized low-density lipoprotein cholesterol (LDL-C).

**Table 1:** Dietary and exercise interventions in children and adolescents with NAFLD

Study	Study population	Type of intervention	Duration of intervention	Completion rate	Results
Dietary-only intervention					
Vos et al. [23]	10 children (mean age 13.3 ± 0.65 years)	Randomized either to a low-fructose diet (*n* = 6) or to a low-fat diet (*n* = 4)	6 months	Not reported	Change in ALT was not significant in either group
Ramon-Krauel et al. [26]	17 children (8–17 years) with obesity (BMI ≥95th percentile for age and sex) and fatty liver	Randomized to either an experimental low-glycemic-load (*n* = 8) or conventional low-fat diet (*n* = 9)	6 months	94% (16/17)	Decrease in liver fat in both groups without intergroup difference. ALT also decreased, in association with liver fat
Jin et al. [25]	24 overweight (BMI z-score ≥ 85th percentile) Hispanic American adolescents (11–18 years)	Randomized to calorie matched fructose-only (*n* = 13) or glucose-only (*n* = 11) beverages	1 month	91.7% (22/24)	No significant change in hepatic fat
Schwimmer et al. [27]	40 adolescent boys (aged 11–16) with NAFLD	Randomized to either a diet low in free sugars (*n* = 20) or a usual diet (*n* = 20)	2 months	100%	Hepatic steatosis reduction significantly greater for the intervention (25% to 17%) vs the control group (21% to 20%) (*p* < 0.001)
Goss et al. [28]	32 children (9–17 years) with obesity (BMI z-score >85th percentile) and NAFLD	Randomized to a moderately carbohydrate-restricted diet (*n* = 16) or a fat-restricted diet (*n* = 16)	2 months	78% (25/32)	Hepatic lipid declined significantly (−6.0 ± 4.7%, *p* < 0.001) only within the carbohydrate-restricted diet group
Exercise-only intervention					
Van der Heijden et al. [33]	15 children (15.6 ± 0.4 years) with obesity (BMI > 95th percentile for age according to CDC growth charts) compared to 14 children with normal weight	Both groups were provided a 30-min intervention twice a week	12 weeks	100%	In children with obesity hepatic fat content decreased from 8.9 ± 3.2 to 5.6 ± 1.8%, *p* < 0.05
Van der Heijden et al. [34]	12 Hispanic adolescents (15.5 ± 0.5 years) with obesity (BMI >95th percentile for age according to CDC growth charts)	Completed a 12 week resistance exercise program	12 weeks	100%	Hepatic fat content remained unchanged
De Piano et al. [35]	28 adolescents (15–19 years) with obesity (BMI >95th percentile of the CDC reference growth charts) and NAFLD	Randomized to aerobic training (*n* = 14) or aerobic plus resistance training (*n* = 14)	1 year	100%	Greater decrease in ALT in the aerobic plus resistance training (–21.84 ± 23.76 IU/L) compared to the aerobic only (– 5.78 ± 9.73 IU/L) training group
Lee et al. [31]	45 adolescent boys (12–18 years) with obesity (≥95th percentile)	Randomly assigned to one of three interventions: aerobic exercise (*n* = 16), resistance exercise (*n* = 16), or a non-exercising control group (*n* = 13)	3 months	96% (43/45)	Significant intrahepatic lipid reduction observed in both exercise groups (−1.9 ± 1.0% and −2.0 ± 1.0%, respectively) compared to the control group (0.9 ± 0.7%)
Lee et al. [32]	44 adolescent girls (12–18 years) with obesity (≥95th percentile)	Randomized to aerobic exercise (*n* = 16), resistance exercise (*n* = 16), or a non-exercising control group (*n* = 12)	3 months	84% (37/44)	Significant reduction in intrahepatic lipid (−1.70 ± 0.74%) observed only in the aerobic and not in the resistance exercise group compared to controls
Labayen et al. [36]	116 children (8–12 years) with hepatic steatosis and overweight/obesity (≥85th or 95th percentile, respectively)	Followed either a lifestyle and psycho-education program (*n* = 57) alone or in combination with supervised exercise (90-min high-intensity aerobic workouts x3/week) (*n* = 59)	22 weeks	87.9% (102/116)	Hepatic fat decreased only in the second compared to the first group (−1.20 ± 0.31% vs. 0.04 ± 0.30%, respectively), regardless of baseline value and any change in adiposity (*p* < 0.01)
Dietary plus exercise intervention					
Vajro et al. [37]	9 children (4.9–11.9 years) with obesity (≥95th percentile)	Followed an individualized program of balanced diet and physical activity	12 months	100%	In all patients ALT significantly decreased (*p* < 0.01) and was normalized after the second month of the intervention
Tazawa et al. [38]	73 children (6–14 years) with obesity (≥95th percentile) and elevated ALT	Treated by a mild regimen for obesity	3 months	100%	Normalization of ALT levels in 54/73 (74%) at the end of the study
Nobili et al. [39]	84 children (3–18.8 years) with overweight or obesity (≥85th or 95th percentile, respectively), elevated aminotransferases and biopsy-proven NAFLD	Underwent a 12-month program of lifestyle advice consisting of diet and physical exercise	12 months	67.9% (57/84)	45/57 patients with normal ALT levels. Mean ALT levels reduced from 62 ± 31 to 33 ± 10 IU/L. Fibrosis improvement in 50/57 children
Wang et al. [45]	76 children/adolescents (10–17 years) with obesity (≥95th percentile for age and sex), high ALT, and steatosis on ultrasound	Randomized to usual care (*n* = 19), to hypocaloric diet and aerobic exercise (*n* = 38), or to vitamin E supplement of 100 IU/day (*n* = 19)	1 month	100%	ALT in group 2 was reduced more significantly than in group 3 (88.58 ± 39.99 vs 63.69 ± 27.05, *p* = 0.040, respectively). No changes detected on ultrasound
Pozzato et al. [40]	25 children (6–14 years) with obesity (≥95th percentile for age and sex) and liver steatosis on MRI	Followed a nutrition-behavior intervention based on balanced diet and physical exercise	1 year	100%	Steatosis declined from 34.6% to 7.7% (*p* < 0.0001). Mean reduction in liver fat fraction was 8.0% (4.0%–12.0%)
Grønbæk et al. [46]	117 children (12.1 ± 1.3 years) with obesity (elevated BMI-SDS), 43% of whom had liver steatosis on ultrasound, and 50% increased ALT (>25 U/L),	Followed a weight loss camp program	10 weeks	100%	Steatosis was reduced from 43% of children to 30% and ALT was normalized in all children
Campos et al. [47]	18 post-pubertal adolescents (15–19 years) with obesity (BMI> 30 kgm^2^ or ≥95th percentile) and NAFLD on ultrasound	Followed nutritional counseling once a week and 1 h of combined aerobic and resistance training, three times a week	1 year	100%	Ultrasound findings of NAFLD decreased from 100 to 33% after intervention. Mean ALT levels decreased from 37.2 ± 22.1 to 27.5 ± 15.0 IU/L, respectively (*p* < 0.05)
Devore et al. [48]	83 children (4–20 years) with chronic ALT elevation,	Were followed up in a gastroenterology clinic with 30-min consultations on nutrition and exercise every three months	1 year	47% (39/83)	Mean ALT decreased from 110 ± 26 IU/L to 74 ± 11 IU/L (*p* < 0.05)
Pacifico et al. [50]	135 children (11.5–12.2 years) with obesity (BMI>95th percentile for age and sex) and NAFLD on ultrasound	followed an intervention program with a hypocaloric diet and a 60-min physical exercise, five days a week	1 year	89% (120/135)	ALT decreased from 54 (45–60) to 37 (34–40) IU/L (*p* < 0.0001). Hepatic fat fraction estimated by MRI in 52 children, decreased from 15.2% (10.4–20.0) to 6.4% (2.5–10.3) (*p* < 0.001)
Sanches et al. [51]	33 adolescents (15–19 years) with obesity (BMI>95^th^ percentile for age and sex), and NAFLD on ultrasound	Followed an interdisciplinary therapy of diet, exercise, and psychological support	1 year	100%	ALT decreased from 27 (20–40) to 21 (16–24) IU/L (*p* < 0.05)
Koot et al. [43]	55 children (8–18 years) with severe obesity (BMI-for-age>35 kgm^2^) and hepatic steatosis diagnosed by Magnetic Resonance Spectroscopy	Non-randomly assigned to inpatient treatment, ambulatory treatment, or usual care	6 months	91% (50/55)	Liver steatosis disappeared in 43, 29 and 22% of patients and ALT normalized in 41, 33 and 6% of patients in each of the three described groups, respectively
Chan et al. [44]	52 adolescents (14–18 years) with intrahepatic triglyceride content ≥5%	Randomly assigned to either a dietitian-led lifestyle modification program (*n* = 26) or a conventional pediatrician-led consultation group (*n* = 26)	52 weeks	80.7% (42/52)	Both groups had reduction in intrahepatic triglyceride content by 2–3% with no intergroup difference (*p* > 0.05)
Lefere et al. [49]	204 patients (14 ± 2.3 years) with obesity (BMI z-score>+2) and NAFLD on ultrasound (in 71.1%) and fibrosis stage (F) ≥2 on transient elastography (in 32.8%)	Followed caloric restriction, physical activity, education, and psychosocial support	12 months	38.7% (79/204)	Fibrosis regressed in all patients with baseline fibrosis at least one stage. ALT decreased from 39 (25–66) to 16 (13–25) IU/L

*ALT* alanine aminotransferase, *NAFLD* Non-alcoholic fatty liver disease

A more restrictive low glycemic diet compared to a low-fat diet was evaluated by Ramon-Krauel et al. [26] in 17 obese children with fatty liver, 8–17 years of age. After six months of follow-up, liver fat along with ALT levels decreased substantially in all participants, without any difference between the two groups. More recently, a study by Schwimmer et al. [27] comprised 40 adolescent boys aged 11 to 16 years with NAFLD, diagnosed by hepatic steatosis >10% and ALT level ≥45 µ/L. The authors used magnetic resonance imaging (MRI) proton density fat fraction (MRI-PDFF) to measure the effect of a diet low in free sugars versus a usual diet on hepatic fat content. The study showed that, after eight weeks, the participants in the low-free sugar diet had a greater reduction in hepatic steatosis compared to the control group. In 2020, the effects of a carbohydrate-restricted vs a fat-restricted diet were evaluated in a study by Goss et al. [28]. More specifically, thirty-two children or adolescents (aged 9–17) with obesity and NAFLD were randomized to a moderately carbohydrate-restricted or fat-restricted diet for 8 weeks. It was shown that hepatic lipid content, measured via MRI, decreased significantly only within the carbohydrate-restricted diet group. In addition, significantly greater decreases in abdominal and total fat mass, and insulin resistance, which are all closely associated with NAFLD, were found in response to the carbohydrate-restricted vs the fat-restricted diet group.

### Physical activity interventions

Exercise is recommended as an important lifestyle modification measure in the management of obesity and NAFLD in children and adolescents. Some studies in adults have shown that exercise alone, even in the absence of dietary changes or weight loss, can decrease hepatic fat content and improve its function [29, 30]. Nevertheless, data on pediatric populations are limited and no studies have investigated the effect of exercise alone on children with biopsy-proven NAFLD (Table 1).

Lee et al. conducted two separate studies, one in boys [31] and another in girls [32], investigating the aerobic and resistance exercise effect on children with obesity and, possibly, NAFLD. In the first study [31], forty-five adolescent boys with obesity were randomly assigned to aerobic exercise, resistance exercise, or no exercise group and were followed for three months, without any caloric intake restriction. Intrahepatic lipid was assessed by MRS. A significant reduction in intrahepatic lipid was observed in both exercise groups, compared to controls. Furthermore, the authors observed that the resistance exercise but not the aerobic exercise group, had a significant improvement in insulin sensitivity. Similarly, in the second study [32], forty four adolescent girls with obesity were randomized into an aerobic or a resistance exercise group, and a third group of no exercise. Aerobic, but not resistance exercise, was effective in reducing liver fat in adolescent girls with obesity, independent of weight loss or calorie restriction. Though, a major limitation of both studies was that, at baseline, very few children had sufficient liver fat to be characterized as having NAFLD, since only 12 boys and five girls had hepatic fat fraction ≥5.0 %.

Another group of investigators, Van Der Heijden et al. also performed two separate studies on children, not based on sex of the participants, but on the type of exercise, namely aerobic [33] or resistance [34] exercise. In the first study evaluating aerobic exercise [33], fifteen children with obesity were compared to 14 children with normal weight. Both groups were provided a 30-minute aerobic exercise program twice a week for 12 weeks, which did not lead to weight loss. It was found that children in the obese group had a decrease in the hepatic fat accumulation from 9% to 6%, measured by MRS. However, no significant change in ALT was observed. In addition, no significant changes were observed in any parameter in lean participants. In the second study on resistance training, twelve Hispanic adolescents with obesity performed a 1-h session exercising all major muscle groups, twice a week, for 12 weeks. Despite improvement in several metabolic parameters, such as hepatic insulin sensitivity and glucose production rate, no changes were observed in hepatic fat content. The authors provided no data on ALT levels.

In a RCT by de Piano et al. [35] the effects of aerobic training with aerobic plus resistance training in adolescents with obesity and fatty liver diagnosed by ultrasound, were compared. After one year of follow-up, it was observed that the aerobic plus resistance training was more effective in improving ALT levels along with other noninvasive biomarkers of metabolic derangement, such as insulin, homeostasis model assessment-insulin resistance (HOMA-IR), adiponectin, and agouti-related peptide. Nonetheless, no follow-up ultrasound was performed.

A more recent nonrandomized study was conducted by Labayen et al. [36]. A total of 116 children with overweight or obesity (10.6 ± 1.1 years) and hepatic steatosis on MRI were recruited. For 22 weeks, participants followed a lifestyle and psycho-education program alone, or in combination with a supervised exercise intervention consisting of 90-min high-intensity aerobic workouts, three times a week. At the end of the intervention, the hepatic fat was re-evaluated by MRI and was found to be reduced only in the exercise group, regardless of baseline value and any change in total body adiposity.

### Dietary plus physical activity interventions

Most of the studies carried out to evaluate lifestyle changes in children and adolescents with NAFLD included both diet and physical activity programs (Table 1). As early as in 1994, Vajro et al. [37] first observed that, in children with obesity, persistent ALT increase, and a “bright” liver on ultrasound, a combined program of nutrition and exercise leading to weight loss could be beneficial. Another early study by Tazawa et al. [38] comprised 73 children aged 6–14 years with obesity and elevated ALT. The authors did not apply a strict dietary program, but they adjusted participants’ diet according to each one’s food preferences and advised that they reduce their daily caloric intake by 20%. In addition, increased physical activity was recommended but without a detailed structured exercise program, for three months. The authors observed weight loss in 36/73 children and ALT normalization in 20/73. This ALT reduction was observed mostly in children with weight reduction (17/20), but, unexpectedly, also in some participants with weight gain (3/20).

Ten years later, in 2006, Nobili et al. [39] published the only study so far comprising children with biopsy-proven NAFLD. In this uncontrolled study, 84 children with overweight/obesity, elevated aminotransferases and NAFLD were enrolled. Increased liver fibrosis was identified in 49 (58.3%) patients at baseline, with obesity and age being independent risk factors of fibrosis. All children followed a 12-month program of lifestyle intervention consisting of one-hour nutritional counseling sessions, the prescription of a balanced diet adjusted to individual preferences, and an exercise program. More specifically, a low calorie diet (25–30 Kcal/kg/d) comprised by 50–60% carbohydrate, 25–30% fat, and 15–20% protein was prescribed. In addition, all participants followed a three-time per week aerobic exercise program with 30–45 min-sessions. Children that completed the 12-month program (57/84) showed a significant decrease in body weight (from mean 60.9 to mean 56 kg), along with a significant ALT reduction (from mean 62 to mean 33 IU/L). Among these 57 patients, 52 had body mass index (BMI) ≥ 85th percentile for age and sex at baseline, of whom, most had an improved ultrasound at the end of the study. More specifically, liver echogenicity on ultrasound completely resolved in 5/52 patients, improved in 41/52 patients, and showed no change in 6/52 patients. Nonetheless, post treatment liver biopsy was not performed in any of the participants.

Three more recent trials comprised patients that were diagnosed with NAFLD by liver MRI or MRS. The first by Pozzato et al. [40] comprised 25 children with obesity, nine of whom (36%) were diagnosed with hepatic steatosis by MRI. Mean ALT levels at baseline were 31 IU/L, which were not very increased, albeit above the upper limit of normal. The latter is 22 IU/L for adolescent girls, and 26 IU/L for adolescent boys according to the National Health and Nutrition Examination Survey for adolescents [41] and more recent studies [42]. Participants followed a 1-year nutrition-behavior intervention based on balanced diet and physical exercise. After the intervention, prevalence of hepatic steatosis dropped down to 7.7% from 34.6% at baseline, while no significant decrease in ALT levels was observed. Regarding body weight, at the end of the study participants had a mean decrease in BMI z-score of 0.26 (0.11–0.41). Importantly, this change was accompanied by a decrease in central adiposity with a waist circumference reduction of 1.46 (0.34–2.60) cm. The other study by Koot et al. [43] evaluated children with NAFLD by MRS. In this study, 55 children (8–18 years) with severe obesity and hepatic steatosis were non-randomly assigned to three intervention groups, namely the inpatient treatment group, the ambulatory treatment group, and the usual care group. After six months, 43, 29 and 22% of children, respectively, had no signs of hepatic steatosis. The respective percentages for serum ALT normalization were 41, 33 and 6%. At the end of the six-month period, the BMI z-score change was greater in the inpatient group (−0.37 [−0.5 to − 0.2]) compared both to the ambulatory (− 0.16 [−0.3 to − 0.04]) and the control group (0.06 [−0.01 to 0.13]). Treatment effects were sustained at 1.5 years follow-up in the first two groups. The authors concluded that the intensity of the intervention (inpatient vs ambulatory) does not significantly increase the treatment success rate. The third study, by Chan et al. [44] also used MRS to identify children with intrahepatic triglyceride content ≥5%. Fifty-two children were randomly assigned to either a dietitian-led lifestyle modification program group or a conventional pediatrician-led consultation group. After 52 weeks of follow-up, a reduction of the intra-hepatic triglyceride content to 2–3% was observed in both groups. Regarding body weight, both groups showed a reduction in BMI z-score at 16 weeks of intervention, but this reduction persisted only for the pediatrician-led group at the end of the study. The authors observed that the more the parents and pediatricians were involved, the longer the results of the intervention were sustained.

Many of the studies involving both diet and exercise interventions comprised children that were diagnosed with NAFLD based on high ALT levels and/or abnormal ultrasound findings. Wang et al. [45] performed an RCT with 76 children/adolescents with obesity, high ALT levels and steatosis on ultrasound that were randomized to a control group without any intervention (Group 1), to a group of children with hypocaloric diet and aerobic exercise (Group 2), or to a group receiving vitamin E supplement of 100 IU/day (Group 3). After one month, aspartate aminotransferase (AST), ALT, HOMA-IR and BMI improved in Groups 2 and 3, but not in Group 1. Between Groups 2 and 3, a greater reduction was observed in the camp group compared to the vitamin E group. Another study by Grønbæk et al. [46] comprised 117 children with obesity, 43% of whom had liver steatosis on ultrasound, and 58% increased LFTs. A 10-week weight loss camp program was implemented, and data were collected from the children at the end of the 10-week period, and 12 months later. A 10% weight loss was observed overall, with hepatic steatosis dropping to 30% along with a decrease in ALT levels in all children. In an uncontrolled study by Campos et al. [47] 18 post-pubertal adolescents with obesity and NAFLD on ultrasound, followed nutritional counseling once a week and 1 h of combined aerobic and resistance training, three times a week. After one year, the mean weight loss observed was 11 ± 7.2 kg. Mean ALT levels decreased from 37.2 ± 22.1 at baseline to 27.5 ± 15.0 IU/L at the end of the study (*p* = 0.05). In addition, the prevalence of NAFLD diagnosed by ultrasound, dropped from 100% to 33%. Devore et al. [48] recruited 83 children with chronic ALT elevation, who were followed up for one year in a gastroenterology clinic with 30-min consultation on nutrition and exercise every three months. Only 39/83 patients (47%) completed the program, showing statistically significant decrease in mean BMI z-score, as well as in both ALT and AST. These results, however, should be interpreted with caution, because of the low percentage of completion rate.

One of the largest studies to date was conducted in a tertiary center for children with severe obesity [49]. Lefere et al. recruited 204 patients (median age, 14 years) with severe obesity (mean BMI z-score, +2.8). NAFLD was diagnosed by ultrasonography in 71.1% of these patients, while 32.8% had presumable fibrosis stage (F) ≥2 in transient elastography. All participants were enrolled in an intensive lifestyle therapy with caloric restriction, physical activity program, education regarding healthy lifestyle, and psychosocial support. After six months, liver fibrosis improved in 75% of patients with baseline fibrosis. After one year, these changes persisted, and in 35 patients, fibrosis resolved completely. Serum ALT levels decreased significantly but changes in ALT were not greater in those with fibrosis regression. More specifically, ALT change in those with resolution of fibrosis was −13 IU/L (+1 to −28) vs −28 IU/L (−7 to −72) in those without resolution of fibrosis (*p* = 0.100). One of the major drawbacks of the study is the low percentage of completion rate, thus its results should also be cautiously interpreted.

Two other studies with combined diet and exercise interventions in children and adolescents, focused mainly on changes in vascular structure and function. However, since they present hepatic outcomes as well, they were also included in this review. The first, by Pacifico et al. [50] enrolled 135 children with obesity and NAFLD based on ultrasound. In addition, 52 children underwent hepatic MRI for steatosis estimation. Fifteen subjects were lost to follow-up. The other 120 followed a 1-year intervention program with a hypocaloric diet and a 60-min physical exercise, five days a week. At the end of the study period, a significant decrease in the BMI-standard deviation score (SDS) was observed (mean −0.34, −0.30 to −0.40), along with a decrease in mean ALT levels from 54 to 37 IU/L, and a significant decrease in hepatic fat content measured by hepatic MRI. The second study by Sanches et al. [51] comprised 131 adolescents with obesity, out of whom, 33 were diagnosed with NAFLD determined by ultrasound. All subjects followed a one-year interdisciplinary therapy of diet, exercise, and psychological support. At the end of the study, a decrease in BMI was observed, from 39.5 to 34.6 kg/m^2^, together with a decrease in the mean ALT levels from 27 to 21 IU/L. However, no follow-up ultrasound was performed.

### Dietary supplements: antioxidants

Vitamin E has been known to have antioxidant, anti-inflammatory, and anti-apoptotic properties. Therefore, it has been studied as a supplement in adult patients with NAFLD and NASH and has been shown to be effective in some studies [15, 52–55], while others showed no improvement, especially regarding the level of fibrosis [14, 56, 57]. However, it should be highlighted that the duration of all relevant studies was possibly short to show improvement in a hard endpoint like hepatic fibrosis [58]. On the other hand, concerns have been raised due to possible adverse effects of the long-term administration of high-dose vitamin E in adults [59]. Therefore, current adult guidelines recommend that vitamin E supplementation may be used in selected patients with NASH and F ≥ 2 for up to 2 years [60].

In pediatric populations with NAFLD, several studies have been conducted to identify the possible effect of vitamin E supplementation (Table 2). A study by Wang et al. [45] compared three groups of children with obesity and NAFLD, aged 10–17, for one month. Group 1 was the control group, group 2 comprised children in a summer camp that followed a strict lifestyle intervention and children in group 3 received oral vitamin E at a dose of 100 mg/d. The study showed that vitamin E supplementation decreased ALT in Group 2 and 3 compared to the control group; however, the reduction in ALT was greater in the children of the lifestyle intervention group than vitamin E group (88.58 ± 39.99 vs 63.69 ± 27.05, *p* = 0.040, respectively). In a large study by Nobili et al. [61] with a longer follow-up period of 24 months, the addition of alpha-tocopherol (a vitamin E form) 600 IU/day plus ascorbic acid (vitamin C) 500 mg/day to lifestyle intervention was investigated in children with biopsy-proven NAFLD. Importantly, the effects of the intervention were evaluated by liver biopsy, looking for the grade of steatosis, hepatocyte ballooning, and lobular inflammation in liver histology at 24 months. Secondary end points included changes in LFTs, body weight, and indices of insulin sensitivity on an oral glucose tolerance test. The study showed that the efficacy of the combination of vitamin E and vitamin C was similar to the efficacy of lifestyle intervention alone, since primary and secondary end points were similar for both groups at the end of the intervention period. A noteworthy finding was that ALT normalized in more children in the placebo compared to the antioxidant group (22/28 vs 13/25, respectively, *p* < 0.05). Another study showed that vitamin E led to significant reduction in ALT (>50 percent from baseline or even normalization) in 38% of children with NASH in a "real world" setting [62]. Furthermore, the combined administration of vitamin E and the antioxidant hydroxytyrosol improved steatosis grade in 40 adolescents with NAFLD compared to the placebo group [63]. In another RCT (TONIC trial) [64], vitamin E supplementation was compared to metformin. More specifically, 173 children and adolescents (aged 8–17 years) with biopsy-confirmed NAFLD, were randomized to daily dosing of 800 IU of vitamin E, 1000 mg of metformin, or placebo for 96 weeks. The study showed that neither vitamin E, nor metformin were superior to placebo regarding the primary outcome, which was a reduction by ≥50% of the baseline ALT level or an ALT level ≤40 IU/L, at every 12-week visit from 48 to 96 weeks of treatment. More specifically, only 15/58 (26%) of the vitamin E group, and 9/57 (16%) of the metformin group met the primary outcome at 96 weeks, compared to 10/58 (17%) of the placebo group (*p* = 0.26 and *p* = 0.83, respectively). Similarly, no statistical significance was identified among the three groups regarding hepatocellular ballooning, NAFLD activity score, and other histological features on follow-up liver biopsy. What is interesting though, is the fact that, among children with NASH, NASH resolved at 96 weeks in more children in the vitamin E group (58%) compared to the placebo (28%; *p* = 0.006) or to the metformin group (41%). Akcam et al. [65] also compared a 6-month metformin or vitamin E supplement in addition to exercise, and nutritional counseling in adolescents with obesity and NAFLD. Metformin treatment was superior to vitamin E in improving metabolic syndrome components. However, no change was observed in ALT levels in any group. Of note, no follow-up ultrasound was performed in this study. Another small study by Vajro et al. [13] allocated 28 children with obesity, NAFLD and high LFTs either to placebo, or to vitamin E supplementation. The interesting finding was that vitamin E supplementation was effective in ALT normalization in those children with minimal adherence to dietary and physical activity recommendations. Indeed, there was a poor compliance to lifestyle interventions (13/28 participants, 46%), compared to the 100% compliance to vitamin E supplementation. The authors suggested that vitamin E could be considered as an effective approach in such patients. Even earlier was a study by Lavine et al. [66] who showed that 4 to 10 months of daily oral vitamin E administration to 11 children with obesity and NASH led to normalization of their AST and ALΤ levels.

**Table 2:** Dietary supplements in children and adolescents with NAFLD

Study	Study population	Type of intervention	Duration of intervention	Completion rate	Results
Antioxidants					
Lavine et al. [66]	11 subjects (<16 years) with obesity (BMI ≥ 95th percentile) and elevated AST, ALT	They were prescribed 400–1200 IU/day oral vitamin E	4–10 months	100%	ALT decreased from 175 ± 106 U/L to 40 ± 26 U/L (*p* < 0.001). Ultrasound findings did not change
Vajro et al. [13]	28 children with obesity (BMI ≥ 95th percentile) and NAFLD	Allocated to group 1 (*n* = 14, 9,88 ± 3,97 years): low-calorie diet with oral placebo, and group 2 (*n* = 14, 10.7 ± 3.45): low-calorie diet plus oral vitamin E	5 months	96.4% (27/28)	ALT levels significantly decreased in both groups (−29.92 and −24.09 U/L, respectively). NAFLD persisted on ultrasound of all children
Wang et al. [45]	76 children/adolescents (10–17 years) with obesity (BMI ≥ 95th percentile for age and sex) and NAFLD	Allocated to Group 1 (*n* = 38) control group, Group 2 (*n* = 19) lifestyle intervention, Group 3 (*n* = 19) oral vitamin E therapy 100 mg/d	1 month	100%	ALT in group 2 was reduced more significantly than in group 3 (88.58 ± 39.99 vs 63.69 ± 27.05, *p* = 0.040, respectively). No changes detected on ultrasound
Nobili et al. [61]	53 children (5.7–18.8 years) with biopsy-proven NAFLD	Following a lifestyle intervention were randomized to alpha-tocopherol 600 IU/day plus ascorbic acid 500 mg/day (*n* = 25) or placebo (*n* = 28)	24 months	37.7% (20/53)	ALT normalized in more children in the placebo compared to the antioxidant group (22/28 vs 13/25, respectively, *p* < 0.05). Histology findings improved in both groups without intragroup difference
Lavine et al. [64]	173 patients (8–17 years) with increased ALT and biopsy-proven NAFLD	Allocated to 800 IU/d vitamin E (*n* = 58), 1000 mg/d metformin (*n* = 57), or placebo (*n* = 58)	24 months	87% (150/173)	ALT change was −35.2 U/L (−56.9 to −13.5) in placebo vs −48.3 U/L (−66.8 to −29.8) in vitamin E (*p* = 0.07) and −41.7 U/L (−62.9 to −20.5) in metformin group (*p* = 0.40). Resolution of NASH was greater in vitamin E than in placebo (25/43 vs 11/39, *p* = 0.006). No significant effect of vitamin E on steatosis and fibrosis
Akcam et al. [65]	57 adolescents (9–17 years) with obesity (BMI ≥ 95th percentile for age and sex), NAFLD on ultrasound	They were divided into the metformin group (850 mg/d) and the vitamin E group (400 U/d), plus an individually tailored diet, exercise, and behavioral therapy	6 months	100%	No significant change was observed in ALT levels in any of the groups
Nobili et al. [63]	80 children/adolescents (13 ± 2.8 years) with biopsy-proven NAFLD	Randomized into oral dose of 7.5 mg hydroxytyrosol and 10 mg vitamin E (*n* = 40) or placebo (*n* = 40)	4 months	87.5% (70/80)	No significant mean ALT decrease between the intervention (47.7 to 42.7 U/L) and the placebo (38.3 to 36.2) groups (*p* = 0.40). Ultrasound findings improved in both with better results in the intervention group, only for severe steatosis (*p* = 0.002)
Yodoshi et al. [62]	73 children (8–19 years) with biopsy-proven NASH	Received vitamin E and were evaluated retrospectively	6–24 months	Not available	Mean ALT decreased from 96 (76–144) to 59 (38–104) U/L (*p* < 0.001)
Kamari et al. [67]	160 children (8–11 years) with obesity (BMI ≥ 95th percentile for age and sex), and NAFLD on ultrasound,	Allocated to four groups: control group, anti-inflammatory-diet group, or ginger- and ginger-with-anti-inflammatory-diet groups	3 months	100%	Significant ALT decrease in the two ginger groups (*p* = 0.004 and <0.001, respectively). Greater steatosis decrease in the ginger-with-anti-inflammatory-diet group (by one degree in 82.5%, and by two degrees in 17.5%)
Probiotics					
Vajro et al. [74]	20 children (10.7 ± 2.1 years) with obesity (BMI ≥ 95th percentile for age and sex), and persistent high ALT (>40 µ/L)	They received either probiotic Lactobacillus rhamnosus strain GG (12 billion CFU/day) or placebo	2 months	100%	Mean ALT decreased more in the intervention group (70.3 ± 34.76 to 40.1 ± 22.37) compared to the control group (63.6 ± 18.47 to 61.6 ± 31.80) (*p* = 0.03)
Alisi et al. [75]	48 children (11 ± 1.5 years) with obesity (BMI > 85th percentile), and biopsy-proven NAFLD	Randomized either to VSL#3 (*n* = 22) or to placebo (*n* = 22) supplementation. A low calorie diet and a moderate exercise program were also prescribed to both groups	2 months	92% (44/48)	No difference in mean ALT level between the two groups. No follow-up biopsy was performed. At baseline, moderate and severe NAFLD were present in 55% and 45% of probiotic group and in 64% and 36% of placebo group. At the end, probability (obtained from an ordinal model) that participants had none, light, moderate or severe fatty liver was 21%, 70%, 9% and 0% in the probiotic group compared to 0%, 7%, 76% and 17% in the placebo group (*p* < 0.001)
Famouri et al. [76]	64 children (10–18 years) with obesity (BMI ≥ 85th percentile) and NAFLD on ultrasound	Allocated to receive probiotic capsule or placebo	3 months	100%	Mean ALT levels decreased from 32.8 ± 19.6 to 23.1 ± 9.9 µ/L in the probiotic group and from 28.9 ± 13.7 to 26.2 ± 12.9 µ/L in the placebo group (*p* = 0.02). Normal liver ultrasound findings in 17 (53.1%) of the intervention and 5 (16.5%) of the placebo group (*p* = 0.008)
PUFAs					
Nobili et al. [84]	60 children (6–16 years) with obesity (BMI ≥ 95th percentile for age and sex), NAFLD on ultrasound ± biopsy-proven NASH	They were randomized to 250 (*n* = 20) or 500 (*n* = 20) mg DHA per day or to placebo (*n* = 20)	6 months	100%	No significant between-DHA group changes in ALT were detected. Both children treated with DHA 250 mg/day (OR = 0.01, 95% CI 0.002 to 0.11, *p* < 0.001) and with DHA 500 mg/day (OR = 0.04, 0.002 to 0.46; *p* = 0.01) decreased severe steatosis more than those treated with placebo. No difference between the DHA groups
Nobili et al. [85]	The three groups of children with NAFLD of the previous study		18 additional months	100%	ALT lower in both DHA groups from 12th month onwards. This result persisted until the end of the study. The decrease in liver fat persisted unchanged at the end of 24 months
Janczyk et al. [86]	76 children (11.1–15.2 years) with overweight/obesity (according to International Obesity Task Force BMI charts) and NAFLD on ultrasound	Randomized either to receive PUFA or placebo	6 months	84.2% (64/76)	No difference between the two groups in the number of patients with decreased ALT by ≥0.3 times the upper normal limit (24 vs 23) or in median ALT (48.5 (31–62) µ/L vs 39 (27–55) µ/L). No differences in ultrasound findings
Boyraz et al. [87]	108 children (9–17 years) with obesity (BMI > 95th percentile for age and sex) and NAFLD	Randomized into the PUFA group receiving 1000 mg daily EPA/DHA supplementation and the placebo group, both with lifestyle intervention	12 months	100%	Frequency of high ALT decreased more in the PUFA group (39.2% to 14.2%) compared to the placebo group (38.4% to 28.8%) (*p* < 0.01). Prevalence of steatosis decreased in 67.8% (38/56) of the patients in the PUFA group compared to 40.4% (21/52) in the control group (*p* = 0.01)
Pacifico et al. [88]	58 children (10.8 ± 2.8 years) with biopsy-proven NAFLD	Randomized into DHA and placebo groups and hepatic fat fraction was estimated by MRI	6 months	87.9% (51/58)	Similar between group changes in ALT. Liver fat on MRI was reduced by 53.4% (95% CI, 33.4–73.4) in the DHA group, vs 22.6% (6.2–39.0) in the placebo group (*p* = 0.040)
Zöhrer et al. [89]	43 children/adolescents (13.2 ± 2.3 years) with biopsy-proven NASH	Assigned to lifestyle modification plus placebo or lifestyle modification plus a mix containing choline, docosahexaenoic acid, and vitamin E	12 months (6 months treatment, 6 months follow-up)	93% (40/43)	ALT was improved in the intervention compared to the control group (53.5 ± 32.6 to 35.3 ± 20.7 vs 51.2 ± 51.6 to 32.5 ± 17.8 µ/L, respectively *p* = 0.04). Severe liver steatosis on ultrasound decreased more in the intervention compared to the placebo group (50 to 5 vs 35 to 15 patients, respectively, *p* = 0.001)
Spahis et al. [90]	20 male children/adolescents (8–18 years) with NAFLD on ultrasound	They received 2 g daily n-3 PUFA supplement	6 months	100%	ALT decreased significantly (52.10 ± 4.45 to 37.75 ± 5.74 µ/L, *p* = 0.0065). Fatty liver index and ALT/AST ratio were also decreased

*ALT* alanine transaminase, *AST* aspartate transaminase, *DHA* Docosahexaenoic acid, *MRI* Magnetic Resonance Imaging, *NAFLD* Non-alcoholic fatty liver disease, *NASH* non-alcoholic steatohepatitis, *PUFA* Polyunsaturated fatty acid

Another antioxidant with anti-inflammatory properties that has been investigated in children with NAFLD is ginger. Indeed, Kamari et al. [67] examined the effect of adding ginger to an anti-inflammatory diet low in processed food in 160 children (8–11 years of age) with obesity and NAFLD. They found that both BMI and ALT levels improved in the ginger and ginger-with-anti-inflammatory-diet groups compared to controls (for BMI *p* = 0.04 in both cases, and for ALT *p* = 0.004 and <0.001, respectively). Similarly, a significant reduction in liver fat accumulation was identified for both intervention groups. This reduction was greater in the ginger-with-anti-inflammatory-diet group (one-degree reduction of steatosis in 82.5% of participants, and two-degree reduction of steatosis in 17.5%).

Among the nutraceutical compounds, curcumin, the most frequently used polyphenol with anti-inflammatory and antifibrotic properties, has been shown to be of value in the context of NAFLD, both in basic and clinical studies [68, 69]. All relevant clinical studies, though, comprised adult patients. There is only one randomized placebo-controlled clinical study with children by Ismail et al [70], which showed that the daily administration of 500 mg curcumin to obese children for 4 weeks, lead to a decrease in their insulin resistance.

### Dietary supplements: probiotics

There is evidence suggesting that gut microbiota may play crucial role in NAFLD development. Indeed, since the liver receives almost 70% of its blood supply from the intestines via the portal circulation, bacteria products, such as lipopolysaccharides and endotoxins can increase hepatic inflammation and oxidative stress [71].

It is, therefore, rational that studies with probiotics and gut microbiota modifiers have been conducted both in adult and pediatric populations with NAFLD (Table 2) [72, 73]. Vajro et al. [74] demonstrated that children with obesity, persisting hypertransaminasemia and ultrasonographic NAFLD that were treated for 8 weeks with the probiotic *Lactobacillus rhamnosus strain GG* had lower ALT levels compared to placebo treated children. Alisi et al. [75] showed that children with biopsy-proven NAFLD that were supplemented with a mixture of eight strains of bacteria had improved liver ultrasound findings after 8 weeks. In another study by Famouri et al. [76] children with obesity and NAFLD that were treated with a daily mixture of four probiotics (*Lactobacillus acidophilus*, *Lactobacillus rhamnosus*, *Bifidobacterium lactis*, and *Bifidobacterium bifidum*), were more likely to decrease ALT levels and normalize ultrasound findings compared to the placebo group. Unfortunately, the small number of the relevant RCTs [74, 75], their small sample sizes, the differences in the probiotic strains used, the short follow-up and the lack of paired liver biopsies render the results insecure. Especially regarding follow-up, it is known that discontinuation of probiotic supplementation is usually followed by a rapid rebound to baseline microbiota composition, rendering studies with longer follow-up necessary [77].

### Dietary supplements: polyunsaturated fatty acids

NASH severity and modified gene expression have been shown to be associated with decreased polyunsaturated fatty acids (PUFAs) hepatocyte content [78]. In addition, epidemiological studies show an inverse relationship between PUFAs intake and the risk of NAFLD and NASH development [79]. Therefore, dietary ω3 PUFA supplements, such as docosahexaenoic (DHA) and eicosapentaenoic acid (EPA), have been investigated for the management of NAFLD in adults. Some clinical trials and meta-analyses support a possible beneficial role of PUFA supplementation in improving LFTs [56, 79–81]. However, other studies did not provide favorable results of PUFAs on hepatic histology of patients with NAFLD [82]. Thus, PUFAs do not seem to be beneficial in adult patients with NAFLD [83].

Regarding studies in pediatric populations, seven trials have been conducted so far (Table 2). Nobili et al. [84] compared the effect of DHA supplementation on liver fat content, in children with biopsy-proven NAFLD. More specifically, 250 or 500 mg DHA were administered to 60 children with NAFLD for 6–24 months and were compared to the placebo group. Children in both DHA groups demonstrated a decrease in liver fat content as detected by ultrasonography after 6 months of treatment. ALT did not significantly change after treatment. In a 24-month extension of this study [85], the difference between DHA and placebo groups persisted. In addition, ALT levels were lower in the DHA 250 mg than placebo group, after the 12th month onwards. However, no follow-up biopsy was performed. Three subsequent RCTs were published on the topic, albeit with conflicting results. More specifically, Janczyk et al. [86] examined 76 children with overweight/obesity and NAFLD that were randomized to receive either omega-3 fatty acids (DHA and EPA, 450−1300 mg/day) or placebo (omega-6 sunflower oil). After six months, no difference was found in ALT levels or the liver steatosis on ultrasound between the two groups. However, AST and gamma-glutamyl transferase (GGT) levels were lower in the intervention compared to the placebo group. Boyraz et al. [87] examined 108 children with obesity and NAFLD, randomized into two groups, one receiving 1000 mg EPA/DHA supplementation daily and the other placebo. Both groups underwent a lifestyle intervention. At the end of the 12-month observation period, both groups showed improvement, but the EPA/DHA group had significantly greater rate of ALT decrease compared to the placebo group (39.2% to 14.2% vs 38.4% to 28.8%, respectively, *p* = 0.01). In addition, the rates of children with steatosis decreased more significantly in the EPA/DHA group (56 patients before to 18 patients after intervention) compared to the control group (52 to 31, respectively). In another study, by Pacifico et al. [88] it was shown that hepatic fat measured by MRI decreased after 6 months of DHA supplementation to children with obesity and biopsy-proven NAFLD compared to the placebo group, despite similar reduction in ALT. Zöhrer et al. [89] also compared placebo vs a mixture of PUFAs with additional antioxidant molecules (namely, DHA with choline and vitamin E) along with lifestyle modification in an RCT with 43 children with biopsy-proven NASH. A significant decrease in severe steatosis in the treatment group (50% to 5%, *p* = 0.001) was observed on ultrasound. Importantly, a follow-up biopsy only in the intervention group showed significant improvement in steatosis, ballooning, and NASH. Finally, Spahis et al. [90] demonstrated that 6 months of 2 g daily n-3 PUFA supplementation in 20 male children/adolescents with NAFLD resulted in decreased Fatty Liver Index, a non-invasive index of steatosis, ALT levels and ALT/AST ratio, indicating beneficial effect on liver steatosis.

Based on the existing studies, definite conclusion on the effect of PUFAs administration in children and adolescents with NAFLD cannot be drawn. Longer-term studies with paired liver biopsies and hard endpoints, i.e., resolution of NASH and/or improvement of hepatic fibrosis are necessary to clarify any effect of PUFA in children/adolescent NAFLD.

## Discussion

The current critical review included all trials that have evaluated the efficacy of lifestyle interventions in children and/or adolescents with MASLD. Such interventions include dietary changes, programs of resistance and/or aerobic exercise, or a combination of both. In addition, studies on dietary supplements, such as vitamin E, probiotics, and PUFAs, were also included. Overall, studies on diet alone have shown some improvement in pediatric MASLD, especially when it is accompanied by weight loss. However, no definite conclusion as to which is the best dietary approach, can be drawn. Data on exercise alone are scarce but suggest some improvement in liver steatosis in children affected by obesity. Most studies have examined the combination of diet and exercise, suggesting a beneficial effect on laboratory or imaging surrogate markers of childhood MASLD. Studies on dietary supplements have shown contradictory results, which are further characterized by high heterogeneity in their endpoints and lack of paired liver biopsies in most of them.

Regarding studies on dietary modifications alone for the management of pediatric MASLD, five trials have been included in this review. All focused on carbohydrate or fat intake reduction, either compared to each other, or compared to a usual diet. Regarding carbohydrate reduction, a special focus on fructose can be observed. Indeed, excessive dietary fructose consumption has been linked to a higher risk of MASLD and hepatic fibrosis development, possibly through increased de novo lipogenesis [91, 92]. Two studies examined whether a low fructose diet can improve MASLD in children, with conflicting results [23, 25]. Other monosaccharides or disaccharides can be involved in MASLD pathogenesis, and a low-free sugar diet was shown to considerably improve hepatic steatosis compared to usual diet [27]. However, no definitive conclusion can be drawn if a carbohydrate- or a fat-restricted diet is more beneficial for children with MASLD, since the results are conflicting [26, 28]. It seems that the main drive of improvement of MASLD is weight loss, while the diet macronutrients and micronutrients seem to be of secondary importance, if any [93]. Indeed, even if MASLD can sometimes be diagnosed in lean subjects [94], obesity is a major risk factor identified in studies in children [95, 96]. Therefore, weight loss, together with the improvement in several of the metabolic syndrome parameters [97], could be, at least in part, responsible for the amelioration of MASLD in obese children after various dietary interventions. Future studies comprising normal weight children with MASLD who do not require weight loss could be of help to determine the best type of diet, although, in lean MASLD the underlying pathophysiology and risk factors may be different (e.g., the genetic factors are more prominent) [98, 99].

Regarding physical activity, indirect evidence on MASLD improvement comes from population studies linking decreased physical activity, sedentary behavior, and inadequate cardiorespiratory fitness with a higher risk of MASLD in children [100, 101]. Physical activity guidelines for the general pediatric population may differ somehow from country to country, but they generally advocate for a daily average of ≥60 min of moderate or vigorous intensity combination of aerobic and resistance exercise, every day of the week [102]. Relevant recommendations have been published by NASPGHAN also for children with MASLD [11]. However, no robust data exist on the possible beneficial effects of exercise on hepatic function in such children. Relevant studies conducted so far and included in this review comprise mostly children with obesity that followed a structured aerobic and/or resistance exercise program and managed to lose weight [31–35]. However, some, but not all, of these children had MASLD at baseline and no trial has been conducted comprising children with biopsy-proven MASLD, more specifically with paired liver biopsy. A more recent trial by Labayen et al. [36] focused specifically on the effects of exercise when added to a family-based lifestyle intervention program in children with obesity and hepatic steatosis. This is the first study showing that exercise, as an adjunct to a multicomponent intervention program, can improve MASLD and hepatic function. More such studies, especially RCTs, are needed to establish the importance of exercise in children with MASLD.

Many of the studies included in this review examined the combined effects of diet and exercise. More specifically, a total of 13 studies were identified, comprising 959 children and adolescents, most of whom were diagnosed with MASLD at baseline [37–40, 43–51]. The diagnosis was mainly based on elevated ALT levels and/or abnormal ultrasonographic findings. Duration of intervention varied between one month and one year and the completion rate was between 38.7% and 100%. The efficacy of interventions was estimated based on ALT level reduction, intrahepatic fat change estimated by ultrasound, MRI, or MRS, or fibrosis determined by transient or magnetic resonance elastography. Follow-up liver biopsy was performed in only one of the studies [39]. Most studies showed an improvement in these parameters, suggesting a beneficial effect of a structured, combined intervention program that leads to weight loss. However, no definite conclusions can be drawn regarding the ideal dietary type, as commented above for the studies with diet alone, or the exact type and duration of exercise. What seems unequivocal though, is the beneficial effect of weight loss on pediatric MASLD. Indeed, in all studies included in this review, weight loss was accompanied by improvement in LFTs and/or by decreased hepatic fat content. Lifestyle interventions have been shown to have good effectiveness in weight loss in children, but they are difficult to implement for long-term [103–105].

Apart from diet and exercise interventions, dietary supplements have gained attention lately as a lifestyle approach for children with MASLD. Vitamin E, having antioxidant, anti-apoptotic, and anti-inflammatory properties, has been evaluated as a supplement in adult patients with MASLD and MASH, with certain benefits [15]. In children with MASLD, eight studies have been conducted to date with vitamin E [13, 45, 61–66]. The number of children included in these studies is considerable (551 in total), but these studies are characterized by heterogeneity, a general lack of paired liver biopsies, and by the fact that vitamin E doses are usually much lower than those proposed for adults with MASLD, so definite conclusion on the effect of vitamin E on pediatric MASLD cannot be drawn. Indeed, vitamin E supplementation was shown to improve only some histological findings in a minority of children. In addition, concerns have been raised by findings of increased hemorrhagic stroke and prostate cancer risk in adults receiving vitamin E in high doses [106, 107], although extrapolation of these findings in children is not easy.

To date, three studies have examined the possible effect of probiotics supplementation to children with MASLD [74–76]. Their results may warrant further studies on probiotics in MASLD, but to date definite conclusion cannot be made. Definite limitation of these studies are their small sample sizes, the differences in the probiotic strains used, and, above all, the short-term follow-up, Finally, seven studies examined the effects of PUFAs to children with MASLD [84–90]. Some, but not all, of these studies showed an improvement in LFTs, e.g., normalization of ALT levels and/or improved ultrasonographic or MRI findings. However, these results should not be overestimated, until larger studies with paired liver biopsies clarify the effect of PUFAs on MASLD histology, if any.

### Limitations of the study

Despite being a comprehensive critical review of the current literature, this study has some limitations, mainly related to the heterogeneity of the data included. More specifically, an inherent difficulty of studies on pediatric MASLD is the way the disease is defined in order, not only, to select the correct population for any given intervention, but also, to reliably evaluate the outcome. Indeed, liver biopsy is the current gold standard for MASLD diagnosis and for severity assessment, namely the presence of hepatic steatosis, inflammation, and, most importantly, fibrosis, which is the main histological predictor of morbidity and mortality [108]. However, because of its invasiveness, biopsy was performed only in a few studies, while several others were based on various laboratory or imaging surrogate markers of the disease, either for the recruitment procedure or for the follow-up [109]. ALT measurement was frequently applied because it is inexpensive and easily available, but it has low sensitivity and specificity in both adults [110] and children [11]. According to the most recent NASPGHAN Clinical Practice Guideline for the Diagnosis and Treatment of Nonalcoholic Fatty Liver Disease in Children [11], ALT increased to more than twice the upper limit, after the exclusion of other causes of fatty liver, in overweight or obese children older than 10 years indicates NAFLD with 88% sensitivity, but only 26% specificity. This means that studies based solely on ALT levels as inclusion criteria were subjected to misclassification bias [111]. Similarly, ALT change as an endpoint evaluating the efficacy of intervention, may not necessarily reflect histological improvement. Newer biomarkers, such as increased fibroblast growth factor 21 [112], and newer techniques, such as high-resolution metabolomics [113], seem to be promising alternatives. However, no studies included in this review used such surrogate markers in pediatric populations with MASLD. Regarding imaging modalities, abdominal ultrasound was extensively used. Having a low cost, good safety profile, and high accessibility, ultrasound remains the imaging technique of choice for fatty liver screening, with a sensitivity and specificity of 84.8% (95% confidence interval; 79.5–88.9), and 93.6% (87.2–97.0), respectively [114, 115]. However, sensitivity and specificity of the ultrasound are lower in obese individuals, i.e., the majority of MASLD patients [116]. MRI, MRS and MRI-PDFF are more accurate methods to evaluate liver fat, but they are more expensive and less accessible than ultrasound [116] and were therefore rarely applied in the studies included. Imaging methods used to evaluate liver fibrosis include transient elastography, share wave elastography, and magnetic resonance elastography, all of which were also rarely used [117]. Further to the above difficulties, the relatively low completion rate observed in several of the included studies (Tables 1 and 2), further decrease the reliability of the studies’ results and make their comparison even more difficult.

## Conclusions

In conclusion, lifestyle modifications, including diet and exercise are considered to be beneficial for children and adolescents with MASLD, and therefore are included in the relevant guidelines [10, 11]. Even if the scientific evidence for these recommendations is not robust, the increasing prevalence of obesity and NAFLD and the associated cardiometabolic risk, in conjunction with the absence of a definite treatment, make these lifestyle measures essential in the management of this vexing pediatric health problem. Certainly, well-organized RCTs that will include children with biopsy-proven MASLD, and paired liver biopsies will contribute to clarify and quantify diet and exercise effects on pediatric MASLD. Regarding the various dietary supplements such as vitamin E, probiotics and PUFAs, existing data do not favor their use in children with MASLD. However, larger, and better methodological studies may shed light on the efficacy of some of them in the future, especially vitamin E.

## References

[1] M.E. Rinella, J.V. Lazarus, V. Ratziu, S.M. Francque, A.J. Sanyal, F. Kanwal et al. A multi-society Delphi consensus statement on new fatty liver disease nomenclature. J. Hepatol. **0**, 45 (2023) 10.1097/HEP.0000000000000696 37983810

[2] E.L. Anderson, L.D. Howe, H.E. Jones, J.P.T. Higgins, D.A. Lawlor, A. Fraser, The prevalence of non-alcoholic fatty liver disease in children and adolescents: a systematic review and meta-analysis. PLoS One **10**, e0140908 (2015) 26512983 10.1371/journal.pone.0140908 PMC4626023

[3] A.E. Feldstein, P. Charatcharoenwitthaya, S. Treeprasertsuk, J.T. Benson, F.B. Enders, P. Angulo, The natural history of non-alcoholic fatty liver disease in children: a follow-up study for up to 20 years. Gut. **58**, 1538–1544 (2009) 19625277 10.1136/gut.2008.171280 PMC2792743

[4] J.B. Schwimmer, P.E. Pardee, J.E. Lavine, A.K. Blumkin, S. Cook, Cardiovascular risk factors and the metabolic syndrome in pediatric nonalcoholic fatty liver disease. Circulation. **118**, 277–283 (2008) 18591439 10.1161/CIRCULATIONAHA.107.739920 PMC2996820

[5] M.L. Geurtsen, S. Santos, J.F. Felix, L. Duijts, M.W. Vernooij, R. Gaillard et al. Liver fat and cardiometabolic risk factors among school‐age children. Hepatology. **72**, 119–129 (2020) 31680281 10.1002/hep.31018 PMC7496381

[6] Y.L. Liao, G.Y. Zhu, C. Chang, Non-alcoholic fatty liver disease increases the risk of cardiovascular disease in young adults and children: a systematic review and meta-analysis of cohort studies. Front. Cardiovasc. Med. **10**, 1291438 (2024) 38268853 10.3389/fcvm.2023.1291438 PMC10806083

[7] E.L. Yu, J.B. Schwimmer, Epidemiology of pediatric nonalcoholic fatty liver disease. Clin. Liver Dis. **17**, 196 (2021) 10.1002/cld.1027 PMC8043694 33868665

[8] A.K. Sahota, W.L. Shapiro, K.P. Newton, S.T. Kim, J. Chung, J.B. Schwimmer, Incidence of nonalcoholic fatty liver disease in children: 2009-2018. Pediatrics. **146**, e20200771 (2020) 33214329 10.1542/peds.2020-0771 PMC7706110

[9] X. Zhang, M. Wu, Z. Liu, H. Yuan, X. Wu, T. Shi et al. Increasing prevalence of NAFLD/NASH among children, adolescents and young adults from 1990 to 2017: a population-based observational study. BMJ Open **11**, e042843 (2021) 33947727 10.1136/bmjopen-2020-042843 PMC8098935

[10] P. Vajro, S. Lenta, P. Socha, A. Dhawan, P. McKiernan, U. Baumann et al. Diagnosis of nonalcoholic fatty liver disease in children and adolescents: position paper of the ESPGHAN Hepatology Committee. J. Pediatr. Gastroenterol. Nutr. **54**, 700–713 (2012) 22395188 10.1097/MPG.0b013e318252a13f

[11] M.B. Vos, S.H. Abrams, S.E. Barlow, S. Caprio, S.R. Daniels, R. Kohli et al. NASPGHAN clinical practice guideline for the diagnosis and treatment of nonalcoholic fatty liver disease in children: recommendations from the Expert Committee on NAFLD (ECON) and the North American Society of Pediatric Gastroenterology, Hepatology and Nutrition (NASPGHAN). J. Pediatr. Gastroenterol. Nutr. **64**, 319–334 (2017) 28107283 10.1097/MPG.0000000000001482 PMC5413933

[12] B.J. Perumpail, A.A. Li, N. John, S. Sallam, N.D. Shah, W. Kwong et al. The role of vitamin E in the treatment of NAFLD. Diseases **6**, 86–96 (2018) 30249972 10.3390/diseases6040086 PMC6313719

[13] P. Vajro, C. Mandato, A. Franzese, E. Ciccimarra, S. Lucariello, M. Savoia et al. Vitamin E treatment in pediatric obesity-related liver disease: a randomized study. J. Pediatr. Gastroenterol. Nutr. **38**, 48–55 (2004) 14676594 10.1097/00005176-200401000-00012

[14] H. El Hadi, R. Vettor, M. Rossato, Vitamin E as a treatment for nonalcoholic fatty liver disease: reality or myth? Antioxidants. **7**(1), 12 (2018) 29337849 10.3390/antiox7010012 PMC5789322

[15] K. Sato, M. Gosho, T. Yamamoto, Y. Kobayashi, N. Ishii, T. Ohashi et al. Vitamin E has a beneficial effect on nonalcoholic fatty liver disease: a meta-analysis of randomized controlled trials. Nutrition. **31**, 923–930 (2015) 26059365 10.1016/j.nut.2014.11.018

[16] M. Rahimlou, H. Ahmadnia, A. Hekmatdoost, Dietary supplements and pediatric non-alcoholic fatty liver disease: Present and the future. World J. Hepatol. **7**, 2597 (2015) 26557952 10.4254/wjh.v7.i25.2597 PMC4635145

[17] Y. Park, S.A. Smith-Warner, X. Zhang, Y.J. Park, H. Kim, H. Park et al. Association between use of vitamin and mineral supplement and non-alcoholic fatty liver disease in hypertensive adults. Sci. Rep. **13**, 1–13 (2023) 37608217 10.1038/s41598-023-40868-1 PMC10444877

[18] L.L. Wang, P.H. Zhang, H.H. Yan, Functional foods and dietary supplements in the management of non-alcoholic fatty liver disease: a systematic review and meta-analysis. Front. Nutr. **10**, 1014010 (2023) 36866059 10.3389/fnut.2023.1014010 PMC9971819

[19] A.F.G. Cicero, A. Colletti, S. Bellentani, Nutraceutical approach to Non-Alcoholic Fatty Liver Disease (NAFLD): the available clinical evidence. Nutrients. **10**, 1153 (2018) 30142943 10.3390/nu10091153 PMC6163782

[20] M.E. Rinella, B.A. Neuschwander-Tetri, M.S. Siddiqui, M.F. Abdelmalek, S. Caldwell, D. Barb et al. AASLD practice guidance on the clinical assessment and management of nonalcoholic fatty liver disease. Hepatology. **77**, 1797–1835 (2023) 36727674 10.1097/HEP.0000000000000323 PMC10735173

[21] Y. Xing, J. Fan, H.J. Wang, H. Wang, Comparison of MAFLD and NAFLD Characteristics in Children. Children. **10**, 560 (2023) 36980118 10.3390/children10030560 PMC10047180

[22] M.J. Page, J.E. McKenzie, P.M. Bossuyt, I. Boutron, T.C. Hoffmann, C.D. Mulrow et al. The PRISMA 2020 statement: an updated guideline for reporting systematic reviews. BMJ **372**, 71 (2021) 10.1136/bmj.n71 PMC8005924 33782057

[23] M.B. Vos, M.B. Weber, J. Welsh, F. Khatoon, D.P. Jones, P.F. Whitington et al. Fructose and oxidized low-density lipoprotein in pediatric nonalcoholic fatty liver disease: a pilot study. Arch. Pediatr. Adolesc. Med. **163**, 674–675 (2009) 19581556 10.1001/archpediatrics.2009.93 PMC4259100

[24] S.S. Gidding, B.A. Dennison, L.L. Birch, S.R. Daniels, M.W. Gilman, A.H. Lichtenstein et al. Dietary recommendations for children and adolescents: a guide for practitioners: consensus statement from the American Heart Association. Circulation. **112**, 2061–2075 (2005) 16186441 10.1161/CIRCULATIONAHA.105.169251

[25] R. Jin, J.A. Welsh, N.A. Le, J. Holzberg, P. Sharma, D.R. Martin et al. Dietary fructose reduction improves markers of cardiovascular disease risk in hispanic-American Adolescents with NAFLD. Nutrients. **6**, 3187 (2014) 25111123 10.3390/nu6083187 PMC4145302

[26] M. Ramon-Krauel, S.L. Salsberg, C.B. Ebbeling, S.D. Voss, R.V. Mulkern, M.M. Apura, et al. A low-glycemic-load versus low-fat diet in the treatment of fatty liver in obese children.Childhood Obes. **9**, 252 (2013). 10.1089/chi.2013.0022 PMC3675832 23705885

[27] J.B. Schwimmer, P. Ugalde-Nicalo, J.A. Welsh, J.E. Angeles, M. Cordero, K.E. Harlow et al. Effect of a low free sugar diet vs usual diet on nonalcoholic fatty liver disease in adolescent boys: a randomized clinical trial. JAMA. **321**, 256–265 (2019) 30667502 10.1001/jama.2018.20579 PMC6440226

[28] A.M. Goss, S. Dowla, M. Pendergrass, A. Ashraf, M. Bolding, S. Morrison et al. Effects of a carbohydrate-restricted diet on hepatic lipid content in adolescents with non-alcoholic fatty liver disease: a pilot, randomized trial. Pediatr. Obes. **15**(7), e12630 (2020) 32128995 10.1111/ijpo.12630

[29] K. Hallsworth, G. Fattakhova, K.G. Hollingsworth, C. Thoma, S. Moore, R. Taylor et al. Resistance exercise reduces liver fat and its mediators in non-alcoholic fatty liver disease independent of weight loss. Gut. **60**, 1278–1283 (2011) 21708823 10.1136/gut.2011.242073 PMC3152868

[30] E. Bacchi, C. Negri, G. Targher, N. Faccioli, M. Lanza, G. Zoppini et al. Both resistance training and aerobic training reduce hepatic fat content in type 2 diabetic subjects with nonalcoholic fatty liver disease (the RAED2 Randomized Trial). Hepatology. **58**, 1287–1295 (2013) 23504926 10.1002/hep.26393

[31] S.J. Lee, F. Bacha, T. Hannon, J.L. Kuk, C. Boesch, S. Arslanian, Effects of aerobic versus resistance exercise without caloric restriction on abdominal fat, intrahepatic lipid, and insulin sensitivity in obese adolescent boys: a randomized, controlled trial. Diabetes. **61**, 2787 (2012) 22751691 10.2337/db12-0214 PMC3478522

[32] S.J. Lee, A.R. Deldin, D. White, Y.M. Kim, I. Libman, M. Rivera-Vega et al. Aerobic exercise but not resistance exercise reduces intrahepatic lipid content and visceral fat and improves insulin sensitivity in obese adolescent girls: a randomized controlled trial. Am. J. Physiol. Endocrinol. Metab. **305**(10), E1222–E1229 (2013) 24045865 10.1152/ajpendo.00285.2013 PMC3840217

[33] G.J. Van Der Heijden, Z.J. Wang, Z.D. Chu, P.J.J. Sauer, M.W. Haymond, L.M. Rodriguez et al. A 12-week aerobic exercise program reduces hepatic fat accumulation and insulin resistance in obese, Hispanic adolescents. Obesity **18**, 384–390 (2010) 19696755 10.1038/oby.2009.274

[34] G.J. Van Der Heijden, Z.J. Wang, Z. Chu, G. Toffolo, E. Manesso, P.J.J. Sauer et al. Strength exercise improves muscle mass and hepatic insulin sensitivity in obese youth. Med. Sci. Sports Exercise **42**, 1973–1980 (2010) 10.1249/MSS.0b013e3181df16d9 PMC2944907 20351587

[35] A. De Piano, M.T. De Mello, P.D.L. Sanches, P.L. Da Silva, R.M.S. Campos, J. Carnier et al. Long-term effects of aerobic plus resistance training on the adipokines and neuropeptides in nonalcoholic fatty liver disease obese adolescents. Eur. J. Gastroenterol. Hepatol. **24**, 1313–1324 (2012) 22932160 10.1097/MEG.0b013e32835793ac

[36] I. Labayen, M. Medrano, L. Arenaza, E. Máz, M. Osés, V. Martínez-Vizcáno et al. Effects of exercise in addition to a family-based lifestyle intervention program on hepatic fat in children with overweight. Diabetes Care **43**, 306–313 (2020) 31227585 10.2337/dc19-0351

[37] P. Vajro, A. Fontanella, C. Perna, G. Orso, M. Tedesco, A. De Vincenzo, Persistent hyperaminotransferasemia resolving after weight reduction in obese children. J. Pediatr. **125**, 239–241 (1994) 8040771 10.1016/S0022-3476(94)70202-0

[38] Y. Tazawa, H. Noguchi, F. Nishinomiya, G. Takada, Effect of weight changes on serum transaminase activities in obese children. Acta Paediatr. Jpn **39**, 210–214 (1997) 9141256 10.1111/j.1442-200X.1997.tb03583.x

[39] V. Nobili, M. Marcellini, R. Devito, P. Ciampalini, F. Piemonte, D. Comparcola et al. NAFLD in children: a prospective clinical-pathological study and effect of lifestyle advice. Hepatology. **44**, 458–465 (2006) 16871574 10.1002/hep.21262

[40] C. Pozzato, E. Verduci, S. Scaglioni, G. Radaelli, M. Salvioni, A. Rovere et al. Liver fat change in obese children after a 1-year nutrition-behavior intervention. J. Pediatr. Gastroenterol. Nutr. **51**, 331–335 (2010) 20562718 10.1097/MPG.0b013e3181d70468

[41] J.B. Schwimmer, W. Dunn, G.J. Norman, P.E. Pardee, M.S. Middleton, N. Kerkar et al. SAFETY study: alanine aminotransferase cutoff values are set too high for reliable detection of pediatric chronic liver disease. Gastroenterology. **138**(4), 1357–1364 (2010) 20064512 10.1053/j.gastro.2009.12.052 PMC2846968

[42] S. Bussler, M. Vogel, D. Pietzner, K. Harms, T. Buzek, M. Penke et al. New pediatric percentiles of liver enzyme serum levels (alanine aminotransferase, aspartate aminotransferase, γ-glutamyltransferase): Effects of age, sex, body mass index, and pubertal stage. Hepatology. **68**, 1319–1330 (2018) 28926121 10.1002/hep.29542

[43] B.G.P. Koot, O.H. Van Der Baan-Slootweg, S. Vinke, A.E. Bohte, C.L.J. Tamminga-Smeulders, P.L.M. Jansen et al. Intensive lifestyle treatment for non-alcoholic fatty liver disease in children with severe obesity: inpatient versus ambulatory treatment. Int. J. Obes. **40**, 51–57 (2016) 10.1038/ijo.2015.175 26315844

[44] D.F.Y. Chan, H.K. So, S.C.N. Hui, R.S.M. Chan, A.M. Li, M.M. Sea et al. Dietitian-led lifestyle modification programme for obese Chinese adolescents with non-alcoholic fatty liver disease: a randomized controlled study. Int. J. Obes. **42**, 1680–1690 (2018) 10.1038/s41366-018-0010-8 29453464

[45] C.L. Wang, L. Liang, J.F. Fu, C.C. Zou, F. Hong, J.Z. Xue et al. Effect of lifestyle intervention on non-alcoholic fatty liver disease in Chinese obese children. World J. Gastroenterol. **14**, 1598 (2008) 18330955 10.3748/wjg.14.1598 PMC2693759

[46] H. Grønbæk, A. Lange, N.H. Birkebæk, P. Holland-Fischer, J. Solvig, A. Hørlyck et al. Effect of a 10-week weight loss camp on fatty liver disease and insulin sensitivity in obese Danish children. J. Pediatr. Gastroenterol. Nutr. **54**, 223–228 (2012) 21760546 10.1097/MPG.0b013e31822cdedf

[47] R.M.S. Campos, A. De Piano, P.L. Da Silva, J. Carnier, P.L. Sanches, F.C. Corgosinho et al. The role of pro/anti-inflammatory adipokines on bone metabolism in NAFLD obese adolescents: effects of long-term interdisciplinary therapy. Endocrine. **42**, 146–156 (2012) 22315014 10.1007/s12020-012-9613-3

[48] S. Devore, R. Kohli, K. Lake, L. Nicholas, K. Dietrich, W.F. Balistreri et al. A multidisciplinary clinical program is effective in stabilizing BMI and reducing transaminase levels in pediatric patients with NAFLD. J. Pediatr. Gastroenterol. Nutr. **57**, 119–123 (2013) 23518484 10.1097/MPG.0b013e318290d138 PMC3696482

[49] S. Lefere, E. Dupont, A. De Guchtenaere, S. Van Biervliet, S. Vande Velde, X. Verhelst et al. Intensive lifestyle management improves steatosis and fibrosis in pediatric nonalcoholic fatty liver disease. Clin. Gastroenterol. Hepatol. **20**, 2317–2326.e4 (2022) 34871812 10.1016/j.cgh.2021.11.039

[50] L. Pacifico, M. Arca, C. Anania, V. Cantisani, M. Di Martino, C. Chiesa, Arterial function and structure after a 1-year lifestyle intervention in children with nonalcoholic fatty liver disease. Nutr. Metab. Cardiovasc. Dis. **23**, 1010–1016 (2013) 23018041 10.1016/j.numecd.2012.08.003

[51] P.L. Sanches, A. De Piano, R.M.S. Campos, J. Carnier, M.T. De Mello, N. Elias et al. Association of nonalcoholic fatty liver disease with cardiovascular risk factors in obese adolescents: the role of interdisciplinary therapy. J. Clin. Lipidol. **8**, 265–272 (2014) 24793347 10.1016/j.jacl.2014.02.007

[52] M.A. Pervez, D.A. Khan, A.U.R. Slehria, A. Ijaz, Delta-tocotrienol supplementation improves biochemical markers of hepatocellular injury and steatosis in patients with nonalcoholic fatty liver disease: a randomized, placebo-controlled trial. Complement. Ther. Med. **52**, 102494 (2020) 32951743 10.1016/j.ctim.2020.102494

[53] E. Magosso, M.A. Ansari, Y. Gopalan, I.L. Shuaib, J.W. Wong, N.A.K. Khan et al. Tocotrienols for normalisation of hepatic echogenic response in nonalcoholic fatty liver: A randomised placebo-controlled clinical trial. Nutr. J. **12**, 1–8 (2013) 24373555 10.1186/1475-2891-12-166 PMC3877967

[54] M.Y. Wang, K. Prabahar, M.A. Găman, J.L. Zhang, Vitamin E supplementation in the treatment on nonalcoholic fatty liver disease (NAFLD): Evidence from an umbrella review of meta-analysis on randomized controlled trials. J. Dig. Dis. **24**, 380–389 (2023) 37503812 10.1111/1751-2980.13210

[55] A. Vadarlis, C. Antza, D.R. Bakaloudi, I. Doundoulakis, G. Kalopitas, M. Samara et al. Systematic review with meta-analysis: the effect of vitamin E supplementation in adult patients with non-alcoholic fatty liver disease. J. Gastroenterol. Hepatol. **36**, 311–319 (2021) 32810309 10.1111/jgh.15221

[56] J. Karedath, H. Javed, F.A. Talpur, B. Lal, A. Kumari, H. Kivan et al. Effect of Vitamin E on clinical outcomes in patients with non-alcoholic fatty liver disease: a meta-analysis. Cureus. **14**(12), e32764 (2022) 36686141 10.7759/cureus.32764 PMC9853086

[57] F. Bril, D.M. Biernacki, S. Kalavalapalli, R. Lomonaco, S.K. Subbarayan, J. Lai et al. Role of Vitamin E for nonalcoholic steatohepatitis in patients with type 2 diabetes: a randomized controlled trial. Diabetes Care **42**, 1481–1488 (2019) 31332029 10.2337/dc19-0167

[58] G. Mintziori, S.A. Polyzos, Emerging and future therapies for nonalcoholic steatohepatitis in adults. Expert Opin. Pharmacother. **17**, 1937–1946 (2016) 27564402 10.1080/14656566.2016.1225727

[59] E.R. Miller, R. Pastor-Barriuso, D. Dalal, R.A. Riemersma, L.J. Appel, E. Guallar, Meta-analysis: high-dosage vitamin E supplementation may increase all-cause mortality. Ann. Intern. Med. **142**(1), 37–46 (2005) 15537682 10.7326/0003-4819-142-1-200501040-00110

[60] G. Marchesini, C.P. Day, J.F. Dufour, A. Canbay, V. Nobili, V. Ratziu et al. EASL-EASD-EASO clinical practice Guidelines for the management of non-alcoholic fatty liver disease. J. Hepatol. **64**, 1388–1402 (2016) 27062661 10.1016/j.jhep.2015.11.004

[61] V. Nobili, M. Manco, R. Devito, V. Di Ciommo, D. Comparcola, M.R. Sartorelli et al. Lifestyle intervention and antioxidant therapy in children with nonalcoholic fatty liver disease: a randomized, controlled trial. Hepatology. **48**, 119–128 (2008) 18537181 10.1002/hep.22336

[62] T. Yodoshi, S. Orkin, A.C. Arce-Clachar, K. Bramlage, W. Su, L. Fei et al. Identifying predictors of response to Vitamin E for the treatment of pediatric nonalcoholic steatohepatitis. J. Parenter. Enteral. Nutr. **44**, 1301–1307 (2020) 10.1002/jpen.1766 PMC7590297 31985850

[63] V. Nobili, A. Alisi, A. Mosca, A. Crudele, S. Zaffina, M. Denaro et al. The antioxidant effects of hydroxytyrosol and Vitamin E on pediatric nonalcoholic fatty liver disease, in a clinical trial: a new treatment? Antioxid. Redox. Signal. **31**, 127–133 (2019) 30588836 10.1089/ars.2018.7704

[64] J.E. Lavine, J.B. Schwimmer, M.L. Van Natta, J.P. Molleston, K.F. Murray, P. Rosenthal et al. Effect of Vitamin E or metformin for treatment of nonalcoholic fatty liver disease in children and adolescents: the TONIC randomized controlled trial. JAMA. **305**, 1659–1668 (2011) 21521847 10.1001/jama.2011.520 PMC3110082

[65] M. Akcam, A. Boyaci, O. Pirgon, S. Kaya, S. Uysal, B.N. Dundar, Therapeutic effect of metformin and vitamin E versus prescriptive diet in obese adolescents with fatty liver. Int. J. Vitam. Nutr. Res. **81**, 398–406 (2011) 22673924 10.1024/0300-9831/a000086

[66] J.E. Lavine, Vitamin E treatment of nonalcoholic steatohepatitis in children: a pilot study. J. Pediatr. **136**, 734–738 (2000) 10839868 10.1016/S0022-3476(00)05040-X

[67] N. Kamari, M. Moradinazar, M. Qasemi, T. Khosravy, M. Samadi, H. Abdolahzad, Combination of the effect of ginger and anti‐inflammatory diet on children with obesity with nonalcoholic fatty liver disease: a randomized clinical trial. Food Sci. Nutr. **11**, 1846 (2023) 37051346 10.1002/fsn3.3218 PMC10084988

[68] N.A. Zabihi, M. Pirro, T.P. Johnston, A. Sahebkar, Is there a role for curcumin supplementation in the treatment of non-alcoholic fatty liver disease? The data suggest yes. Curr. Pharm. Des. **23**, 969–982 (2017) 27748192 10.2174/1381612822666161010115235

[69] M. Guariglia, F. Saba, C. Rosso, E. Bugianesi, Molecular mechanisms of curcumin in the pathogenesis of metabolic dysfunction associated steatotic liver disease. Nutrients. **15**, 5053–5060 (2023) 38140312 10.3390/nu15245053 PMC10745597

[70] N.M.I. Nagwa, R. Samer, A.A. El Baky, W.K. Hashem, I. Alshaymaa, Effect of oral curcumin administration on insulin resistance, serum resistin and fetuin-A in obese children: Randomized placebo-controlled study. Res. J. Pharm. Biol. Chem. Sci. **5**, 887–896 (2014)

[71] J. Aron-Wisnewsky, M.V. Warmbrunn, M. Nieuwdorp, K. Clément, Nonalcoholic fatty liver disease: modulating gut microbiota to improve severity? Gastroenterology. **158**, 1881–1898 (2020) 32044317 10.1053/j.gastro.2020.01.049

[72] V. Nobili, S. Cucchiara, The use of probiotics in pediatric nonalcoholic fatty liver disease: teachable moment or missed opportunity? J. Pediatr. Gastroenterol. Nutr. **64**, 336–337 (2017) 27749611 10.1097/MPG.0000000000001431

[73] L. Liu, P. Li, Y. Liu, Y. Zhang, Efficacy of probiotics and synbiotics in patients with nonalcoholic fatty liver disease: a meta-analysis. Dig. Dis. Sci. **64**, 3402–3412 (2019) 31203554 10.1007/s10620-019-05699-z

[74] P. Vajro, C. Mandato, M.R. Licenziati, A. Franzese, D.F. Vitale, S. Lenta et al. Effects of Lactobacillus rhamnosus strain GG in pediatric obesity-related liver disease. J. Pediatr. Gastroenterol. Nutr. **52**, 740–743 (2011) 21505361 10.1097/MPG.0b013e31821f9b85

[75] A. Alisi, G. Bedogni, G. Baviera, V. Giorgio, E. Porro, C. Paris et al. Randomised clinical trial: the beneficial effects of VSL#3 in obese children with non-alcoholic steatohepatitis. Aliment Pharmacol. Ther. **39**, 1276–1285 (2014) 24738701 10.1111/apt.12758 PMC4046270

[76] F. Famouri, Z. Shariat, M. Hashemipour, M. Keikha, R. Kelishadi, Effects of probiotics on nonalcoholic fatty liver disease in obese children and adolescents. J. Pediatr. Gastroenterol. Nutr. **64**, 413–417 (2017) 28230607 10.1097/MPG.0000000000001422

[77] C.A. Lozupone, J.I. Stombaugh, J.I. Gordon, J.K. Jansson, R.Knight, Diversity, stability and resilience of the human gut microbiota. Nature. **489**, 220 (2012). 22972295 10.1038/nature11550 PMC3577372

[78] B.M. Arendt, E.M. Comelli, D.W.L. Ma, W. Lou, A. Teterina, T. Kim et al. Altered hepatic gene expression in nonalcoholic fatty liver disease is associated with lower hepatic n-3 and n-6 polyunsaturated fatty acids. Hepatology. **61**, 1565–1578 (2015) 25581263 10.1002/hep.27695

[79] X.T. Lu, Y.D. Wang, T.T. Zhu, H.L. Zhu, Z.Y. Liu, Dietary fatty acids and risk of non-alcoholic steatohepatitis: a national study in the United States. Front. Nutr. **9**, 952451 (2022) 35958253 10.3389/fnut.2022.952451 PMC9360798

[80] D.B. Jump, K.A. Lytle, C.M. Depner, S. Tripathy, Omega-3 polyunsaturated fatty acids as a treatment strategy for nonalcoholic fatty liver disease. Pharmacol. Ther. **181**, 108–125 (2018) 28723414 10.1016/j.pharmthera.2017.07.007 PMC5743581

[81] K. Musa-Veloso, C. Venditti, H.Y. Lee, M. Darch, S. Floyd, S. West et al. Systematic review and meta-analysis of controlled intervention studies on the effectiveness of long-chain omega-3 fatty acids in patients with nonalcoholic fatty liver disease. Nutr. Rev. **76**, 581–602 (2018) 29917092 10.1093/nutrit/nuy022 PMC6367993

[82] A.J. Sanyal, M.F. Abdelmalek, A. Suzuki, O.W. Cummings, M. Chojkier, No significant effects of ethyl-eicosapentanoic acid on histologic features of nonalcoholic steatohepatitis in a phase 2 trial. Gastroenterology. **147**(2), 377–84.e1 (2014) 24818764 10.1053/j.gastro.2014.04.046

[83] S.A. Polyzos, E.S. Kang, C. Boutari, E.J. Rhee, C.S. Mantzoros, Current and emerging pharmacological options for the treatment of nonalcoholic steatohepatitis. Metabolism. **111S**, 154203 (2020) 32151660 10.1016/j.metabol.2020.154203

[84] V. Nobili, G. Bedogni, A. Alisi, A. Pietrobattista, P. Risé, C. Galli et al. Docosahexaenoic acid supplementation decreases liver fat content in children with non-alcoholic fatty liver disease: double-blind randomised controlled clinical trial. Arch. Dis. Child **96**, 350–353 (2011) 21233083 10.1136/adc.2010.192401

[85] V. Nobili, A. Alisi, C. Della Corte, P. Risé, C. Galli, C. Agostoni et al. Docosahexaenoic acid for the treatment of fatty liver: randomised controlled trial in children. Nutr. Metab. Cardiovasc. Dis. **3**, 1066–1070 (2013) 10.1016/j.numecd.2012.10.010 23220074

[86] W. Janczyk, D. Lebensztejn, A. Wierzbicka-Rucińska, A. Mazur, J. Neuhoff-Murawska, P. Matusik et al. Omega-3 Fatty acids therapy in children with nonalcoholic Fatty liver disease: a randomized controlled trial. J. Pediatr. **166**, 1358–1363.e3 (2015) 25771388 10.1016/j.jpeds.2015.01.056

[87] M. Boyraz, Ö. Pirgon, B. Dündar, F. Çekmez, N. Hatipoğlu, Long-term treatment with n-3 polyunsaturated fatty acids as a monotherapy in children with nonalcoholic fatty liver disease. J. Clin. Res. Pediatr. Endocrinol. **7**, 121–127 (2015) 26316434 10.4274/jcrpe.1749 PMC4563183

[88] L. Pacifico, E. Bonci, M. Di Martino, P. Versacci, G. Andreoli, L.M. Silvestri et al. A double-blind, placebo-controlled randomized trial to evaluate the efficacy of docosahexaenoic acid supplementation on hepatic fat and associated cardiovascular risk factors in overweight children with nonalcoholic fatty liver disease. Nutr. Metab. Cardiovasc. Dis. **25**, 734–741 (2015) 26026214 10.1016/j.numecd.2015.04.003

[89] E. Zöhrer, A. Alisi, J. Jahnel, A. Mosca, C. Della Corte, A. Crudele et al. Efficacy of docosahexaenoic acid-choline-vitamin E in paediatric NASH: a randomized controlled clinical trial. Appl. Physiol. Nutr. Metab. **42**, 948–954 (2017) 28511023 10.1139/apnm-2016-0689

[90] S. Spahis, F. Alvarez, N. Ahmed, J. Dubois, R. Jalbout, M. Paganelli et al. Non-alcoholic fatty liver disease severity and metabolic complications in obese children: impact of omega-3 fatty acids. J. Nutr. Biochem. **58**, 28–36 (2018) 29864682 10.1016/j.jnutbio.2018.03.025

[91] M.F. Abdelmalek, A. Suzuki, C. Guy, A. Unalp-Arida, R. Colvin, R.J. Johnson et al. Increased fructose consumption is associated with fibrosis severity in patients with nonalcoholic fatty liver disease. Hepatology. **51**, 1961–1971 (2010) 20301112 10.1002/hep.23535 PMC2922495

[92] X. Ouyang, P. Cirillo, Y. Sautin, S. McCall, J.L. Bruchette, A.M. Diehl et al. Fructose consumption as a risk factor for non-alcoholic fatty liver disease. J. Hepatol. **48**, 993–999 (2008) 18395287 10.1016/j.jhep.2008.02.011 PMC2423467

[93] S.A. Polyzos, J. Kountouras, C.S. Mantzoros, Obesity and nonalcoholic fatty liver disease: from pathophysiology to therapeutics. Metabolism. **92**, 82–97 (2019) 30502373 10.1016/j.metabol.2018.11.014

[94] A.Y. Wang, J. Dhaliwal, M. Mouzaki, Lean non-alcoholic fatty liver disease. Clin. Nutr. **38**, 975–981 (2019) 30466956 10.1016/j.clnu.2018.08.008

[95] Q. Pang, J.Y. Zhang, S.D. Song, K. Qu, X.S. Xu, S.S. Liu et al. Central obesity and nonalcoholic fatty liver disease risk after adjusting for body mass index. World J. Gastroenterol. **21**, 1650 (2015) 25663786 10.3748/wjg.v21.i5.1650 PMC4316109

[96] V. Cuzmar, G. Alberti, R. Uauy, A. Pereira, C. García, F. De Barbieri et al. Early obesity: risk factor for fatty liver disease. J. Pediatr. Gastroenterol. Nutr. **70**, 93–98 (2020) 31880680 10.1097/MPG.0000000000002523

[97] A. Serbis, V. Giapros, A. Galli-Tsinopoulou, E. Siomou, Metabolic syndrome in children and adolescents: is there a universally accepted definition? Does it Matter? Metab. Syndr. Relat. Disord. **18**, 462–470 (2020) 32795106 10.1089/met.2020.0076

[98] J.K. DiStefano, G.S. Gerhard, NAFLD in normal weight individuals. Diabetol. Metab. Syndr. **14**, 45 (2022) 35331317 10.1186/s13098-022-00814-z PMC8944050

[99] E.J. Kim, H.J. Kim, Nonalcoholic fatty liver disease in obese and nonobese pediatric patients. Korean J. Pediatr. **62**, 30 (2019) 30304905 10.3345/kjp.2018.06786 PMC6351803

[100] J.L. Trilk, A. Ortaglia, S.N. Blair, M. Bottai, T.S. Church, R.R. Pate, Cardiorespiratory fitness, waist circumference, and alanine aminotransferase in youth. Med. Sci. Sports Exercise **45**, 722–727 (2013) 10.1249/MSS.0b013e31827aa875 PMC3605269 23190589

[101] L. Gerber, M. Otgonsuren, A. Mishra, C. Escheik, A. Birerdinc, M. Stepanova et al. Non-alcoholic fatty liver disease (NAFLD) is associated with low level of physical activity: a population-based study. Aliment Pharmacol. Ther. **36**, 772–781 (2012) 22958053 10.1111/apt.12038

[102] A.M. Parrish, M.S. Tremblay, S. Carson, S.L.C. Veldman, D. Cliff, S. Vella et al. Comparing and assessing physical activity guidelines for children and adolescents: a systematic literature review and analysis. Int. J. Behav. Nutr. Phys. Activity **17**, 1–22 (2020) 10.1186/s12966-020-0914-2 PMC7011603 32041635

[103] B. Li, S. Gao, W. Bao, M. Li, Effectiveness of lifestyle interventions for treatment of overweight/obesity among children in China: a systematic review and meta-analysis. Front. Endocrinol. **13**, 972954 (2020) 10.3389/fendo.2022.972954 PMC9659900 36387871

[104] S.L. Samuels, P. Hu, K.R. Maciejewski, F. Li, J. Dziura, M. Savoye et al. Real-world effectiveness of the Bright Bodies healthy lifestyle intervention for childhood obesity. Obesity. **31**, 203–213 (2023) 36502287 10.1002/oby.23627 PMC9780185

[105] A. Ek, M. Brissman, K. Nordin, K. Eli, P. Nowicka, A long-term follow-up of treatment for young children with obesity: a randomized controlled trial. Int. J. Obes. **47**, 1152–1160 (2023) 10.1038/s41366-023-01373-7 PMC10599998 37723272

[106] L. E, B. J, Y. S, S. P, P. J, A. JM, et al. Effects of long-term vitamin E supplementation on cardiovascular events and cancer: a randomized controlled trial. JAMA. **293**, 1338–1347 (2005) 15769967 10.1001/jama.293.11.1338

[107] E.L. Abner, F.A. Schmitt, M.S. Mendiondo, J.L. Marcum, R.J. Kryscio, Vitamin E and all-cause mortality: a meta-analysis. Curr. Aging Sci. **4**, 158 (2011) 21235492 10.2174/1874609811104020158 PMC4030744

[108] E. Makri, A. Goulas, S.A. Polyzos, Epidemiology, pathogenesis, diagnosis and emerging treatment of nonalcoholic fatty liver disease. Arch. Med. Res. **52**, 25–37 (2021) 33334622 10.1016/j.arcmed.2020.11.010

[109] M. Mouzaki, S.C. Ling, R.A. Schreiber, B.M. Kamath, Management of pediatric nonalcoholic fatty liver disease by academic hepatologists in canada: a nationwide survey. J. Pediatr. Gastroenterol. Nutr. **65**, 380–383 (2017) 28333768 10.1097/MPG.0000000000001581

[110] S. Verma, D. Jensen, J. Hart, S.R. Mohanty, Predictive value of ALT levels for non-alcoholic steatohepatitis (NASH) and advanced fibrosis in non-alcoholic fatty liver disease (NAFLD). Liver Int. **33**, 1398–1405 (2013) 23763360 10.1111/liv.12226

[111] J.B. Schwimmer, K.P. Newton, H.I. Awai, L.J. Choi, M.A. Garcia, L.L. Ellis et al. Paediatric gastroenterology evaluation of overweight and obese children referred from primary care for suspected non-alcoholic fatty liver disease. Aliment Pharmacol. Ther. **38**, 1267–1277 (2013) 24117728 10.1111/apt.12518 PMC3984047

[112] L. He, L. Deng, Q. Zhang, J. Guo, J. Zhou, W. Song et al. Diagnostic value of CK-18, FGF-21, and related biomarker panel in nonalcoholic fatty liver disease: a systematic review and meta-analysis. Biomed. Res. Int. **2017**, 9729107 (2017) 28326329 10.1155/2017/9729107 PMC5343245

[113] R.D. Khusial, C.E. Cioffi, S.A. Caltharp, A.M. Krasinskas, A. Alazraki, J. Knight-Scott et al. Development of a plasma screening panel for pediatric nonalcoholic fatty liver disease using metabolomics. Hepatol. Commun. **3**, 1311 (2019) 31592078 10.1002/hep4.1417 PMC6771165

[114] R. Hernaez, M. Lazo, S. Bonekamp, I. Kamel, F.L. Brancati, E. Guallar et al. Diagnostic accuracy and reliability of ultrasonography for the detection of fatty liver: a meta-analysis. Hepatology. **54**, 1082 (2011) 21618575 10.1002/hep.24452 PMC4197002

[115] C.M. Lee, E.L. Yoon, A. Nakajima, M. Yoneda, H. Toyoda, S. Yasuda et al. A reappraisal of the diagnostic performance of B-Mode ultrasonography for mild liver steatosis. Am. J. Gastroenterol. **118**, 840–847 (2023) 36305695 10.14309/ajg.0000000000002020

[116] S.A. Polyzos, C.S. Mantzoros, Necessity for timely noninvasive diagnosis of nonalcoholic fatty liver disease. Metabolism. **63**, 161–167 (2014) 24290839 10.1016/j.metabol.2013.10.010

[117] Q. Yu, Y. Liu, P. Hu, F. Gao, G. Huang, Performance of imaging techniques in non-invasive diagnosis of non-alcoholic fatty liver disease in children: a systematic review and meta-analysis. Front. Pediatr. **10**, 837116 (2022) 35899133 10.3389/fped.2022.837116 PMC9311375

